# Development and characterization of Nb_3_Sn/Al_2_O_3_ superconducting multilayers for particle accelerators

**DOI:** 10.1038/s41598-021-87119-9

**Published:** 2021-04-08

**Authors:** Chris Sundahl, Junki Makita, Paul B. Welander, Yi-Feng Su, Fumitake Kametani, Lin Xie, Huimin Zhang, Lian Li, Alex Gurevich, Chang-Beom Eom

**Affiliations:** 1 Department of Materials Science and Engineering, University of Wisconsin-Madison, Madison, WI 53706 USA; 2 Physics Department and Center for Accelerator Science, Old Dominion University, Norfolk, VA 23529 USA; 3 SLAC National Accelerator Laboratory, Menlo Park, CA 94025 USA; 4 Applied Superconductivity Center, National High Magnetic Field Laboratory, Florida State University, Tallahassee, FL 32310 USA; 5 Department of Mechanical Engineering, FAMU-FSU College of Engineering, Tallahassee, FL 32310 USA; 6 Department of Physics, Southern University of Science and Technology, Shenzhen, 518055 China; 7 Department of Physics and Astronomy, West Virginia University, Morgantown, WV 26506 USA

**Keywords:** Superconducting properties and materials, Surfaces, interfaces and thin films

## Abstract

Superconducting radio-frequency (SRF) resonator cavities provide extremely high quality factors > 10^10^ at 1–2 GHz and 2 K in large linear accelerators of high-energy particles. The maximum accelerating field of SRF cavities is limited by penetration of vortices into the superconductor. Present state-of-the-art Nb cavities can withstand up to 50 MV/m accelerating gradients and magnetic fields of 200–240 mT which destroy the low-dissipative Meissner state. Achieving higher accelerating gradients requires superconductors with higher thermodynamic critical fields, of which Nb_3_Sn has emerged as a leading material for the next generation accelerators. To overcome the problem of low vortex penetration field in Nb_3_Sn, it has been proposed to coat Nb cavities with thin film Nb_3_Sn multilayers with dielectric interlayers. Here, we report the growth and multi-technique characterization of stoichiometric Nb_3_Sn/Al_2_O_3_ multilayers with good superconducting and RF properties. We developed an adsorption-controlled growth process by co-sputtering Nb and Sn at high temperatures with a high overpressure of Sn. The cross-sectional scanning electron transmission microscope images show no interdiffusion between Al_2_O_3_ and Nb_3_Sn. Low-field RF measurements suggest that our multilayers have quality factor comparable with cavity-grade Nb at 4.2 K. These results provide a materials platform for the development and optimization of high-performance SIS multilayers which could overcome the intrinsic limits of the Nb cavity technology.

## Introduction

**Figure 1:**
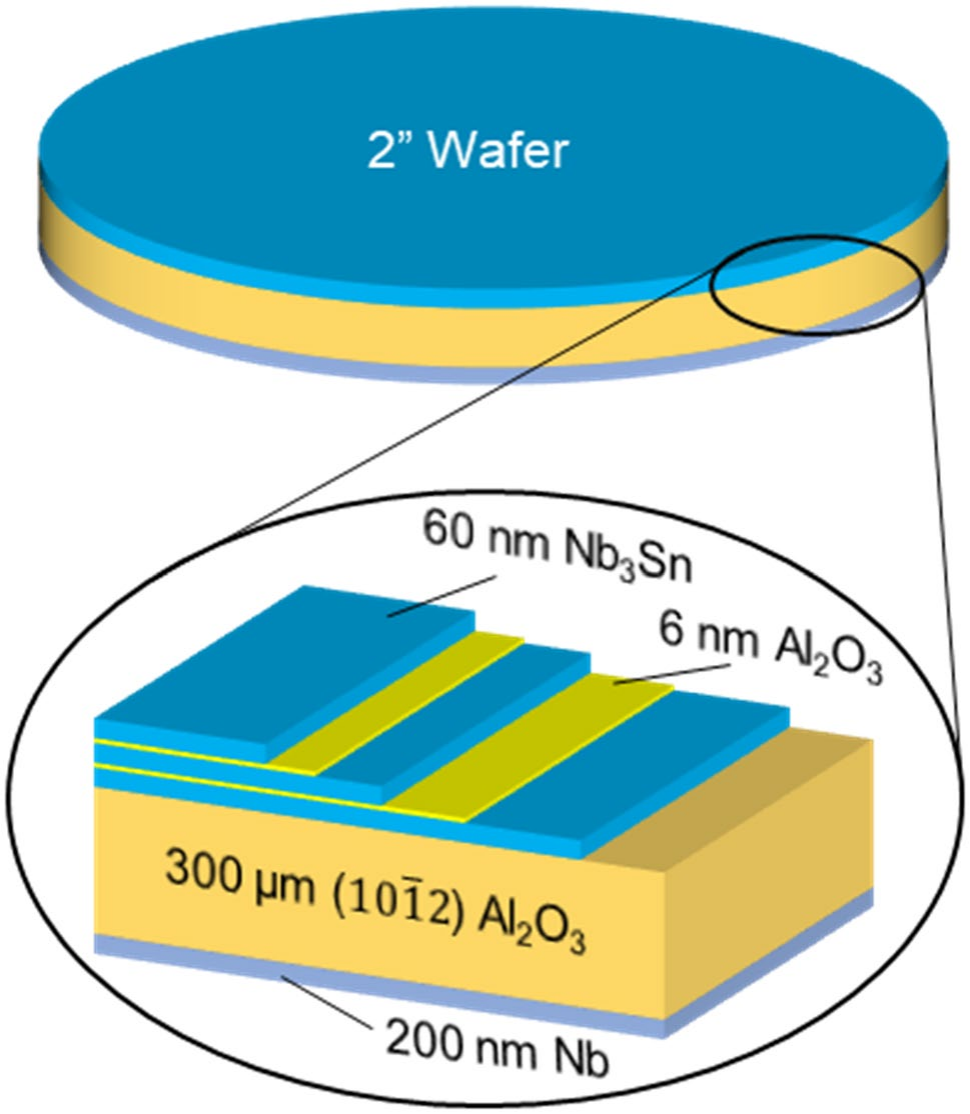
Schematic of Nb_3_Sn/Al_2_O_3_ multilayer heterostructures on Al_2_O_3_ wafer. Back side of Al_2_O_3_ wafer is coated with a thick Nb film.

For decades, Nb has been the material of choice for the radio-frequency superconducting (SRF) resonators for high-energy particle accelerators. Technological advances have resulted in the development of Nb cavities which can exhibit extremely high quality factors Q > 10^10^ @ 1–2 GHz and 2 K while sustaining accelerating gradients up to 50 MV/m^1–3^. Such exemplary performance and low RF losses can only be achieved if the cavities operate in a Meissner state which can persist up to the maximum magnetic field at the inner cavity surface reaches the superheating field B_s_ = 240 mT^1–3^. At B = B_s_ the low-dissipative Meissner state becomes absolutely unstable with respect to dissipative penetration of vortices, causing an explosive increase of RF power and thermal quench of the cavity. The state-of-the-art Nb cavities can already operate at the peak magnetic field close to B_s_, thus, increasing accelerating gradients beyond the intrinsic limits of Nb requires materials with higher B_s_. There are many such materials but all of them are type-II superconductors with lower critical field B_*c*1_ smaller than ${B}_{c1}\approx 170{-}180$ mT of Nb which makes high-B_s_ superconductors prone to detrimental penetration of vortices at low fields^4,5^. To overcome this problem, it was proposed to nanostructure the inner surface of Nb cavities by coating it with multilayers of thin superconductors (S) separated by dielectric insulating (I) layers (Fig. 1)^6^. Here the S-layer material has a superheating field B_s_ higher than B_s0_ of Nb, whereas the thickness *d* of S layers is smaller than the London penetration depth $\lambda$, and the thickness of I layers can be a few nm to suppress the interlayer Josephson coupling. Such SIS structures greatly increase barriers for penetration of vortices in the bulk of the cavity which could potentially withstand the RF fields limited by the superheating field of S-layer. For instance, using Nb_3_Sn with ${B}_{s}=480$ mT could nearly double the maximum accelerating gradient as compared to the best Nb cavities. The multilayer approach is based on the lack of thermodynamically stable parallel vortices in thin decoupled S screens at $B<{B}_{c1}$ where ${B}_{c1}$ is strongly enhanced in films with $d<\lambda$^6–10^. Because the inner surface of the Nb cavity is partially screened by multilayers, both Q(H) and the breakdown field can be increased due to lower surface resistance R_s_ and higher H_c_ of the layer material^6^.

The multilayer coating, which opens up a principal opportunity to break the Nb monopoly in SRF cavities, has been tested by several groups using MgB_2_, Nb_3_Sn, NbN, NbTiN, and dirty Nb as coating materials. These experiments have shown an increase of the dc field onset of penetration of vortices on Nb surfaces coated with different SIS structures^4,11–19^, although such key SRF characteristics as the surface resistance and quality factors of SIS multilayers under high-amplitude RF fields have been investigated to a much lesser extent. The first results on low-field Q measurements on NbN/MgO multilayers^13,19^ have shown that SIS multilayers can have lower R_s_ than bulk Nb. However, the SRF performance of Nb_3_Sn, the current material of choice for the next generation coating material^20^, has not yet been investigated in SIS structures. The development of SIS structures requires overcoming many materials science and technological challenges to achieve good superconducting properties and SRF performance while providing optimal stoichiometry and morphology of the layers and the interfaces and transparency of grain boundaries to extremely high RF current densities. In this work we report results on growth and characterizations of Nb_3_Sn/Al_2_O_3_ multilayers which exhibit good superconducting properties and low-field SRF performance on par with the cavity-grade Nb.

## Results and discussion

### Multilayer growth

We developed a technique of high-temperature confocal sputtering of Nb and Sn from elemental targets to grow stoichiometric Nb_3_Sn multilayers with Al_2_O_3_ interlayers. Details are given in the Supplemental Information. Thin films and multilayers of different thicknesses were grown on different sapphire single crystal substrates for the subsequent characterizations. For instance, 60 nm thick Nb_3_Sn films were grown on 10 × 10 mm sapphire substrates for transport, scanning tunneling spectroscopy and electron microscopy characterizations. For RF tests, we grew Nb_3_Sn/Al_2_O_3_ multilayers on 2″ diameter sapphire wafers (R-plane, 300 μm thick). These multilayers had up to three 60 nm Nb_3_Sn layers separated by 6 nm Al_2_O_3_. The thickness of the Nb_3_Sn layers was chosen to be smaller than the London penetration depth^5,6^. A 200 nm thick Nb film was deposited on the backside of the wafers to prevent leakage of RF field during cavity measurements. The geometry of multilayer samples used in our RF measurements of quality factors is shown in Fig. 1.

**Figure 2:**
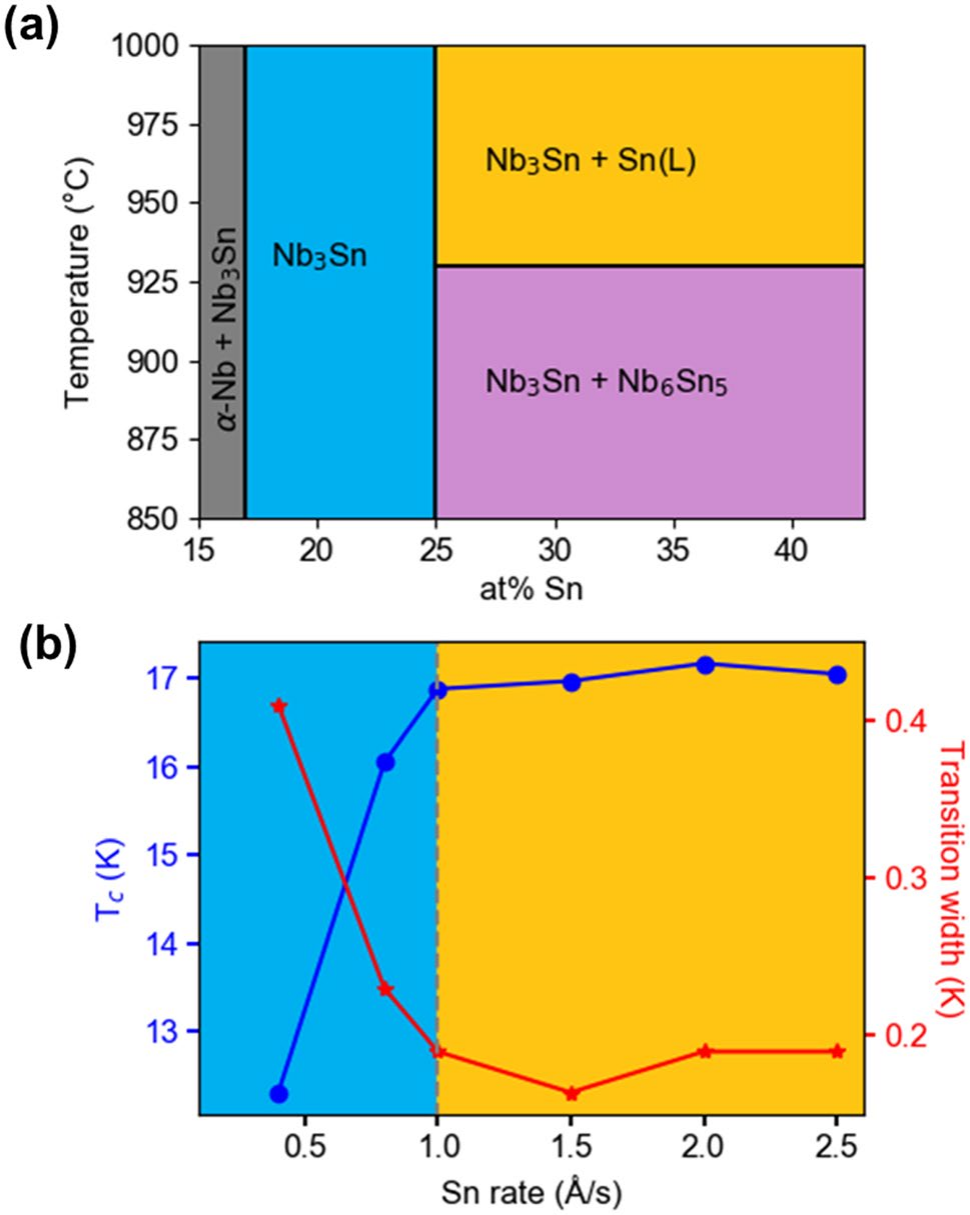
Connecting the Nb-Sn phase diagram to electrical properties and process window. (**a**) Relevant adsorption-controlled growth regime (orange) of Nb-Sn phase diagram. Nb_3_Sn spans 17–25% Sn, and the desired Nb_3_Sn + liquid Sn field lies above 930 °C and 25% Sn. (**b**) T_c_, ΔT_c_, vs the Sn flux of 60 nm thick Nb_3_Sn single layer thin films on Al_2_O_3_ substrates. T_c_ levels off above 1.0 Å/s Sn, corresponding to the adsorption-controlled growth window and the film composition reach 25% Sn.

The Nb-Sn phase diagram contains several line compounds. For instance, Nb_3_Sn and Nb_6_Sn_5_ coexist in the region marked in Fig. 2a. Here a low-T_c_ Nb_6_Sn_5_ phase is clearly undesirable in these films^21^. Within the Nb_3_Sn phase region extending from 17 to 25% Sn, the critical temperature T_c_ degrades steeply as stoichiometry moves away from a 3:1 ratio^22^. These two conditions demand that Nb_3_Sn films should contain 25% of Sn. This was accomplished by providing processing conditions reflecting the field in the upper right of the phase diagram in Fig. 2a, a two-phase region containing only stoichiometric Nb_3_Sn and liquid Sn. Films were grown by confocal sputtering of Nb and Sn from elemental targets. By providing a large over-pressure of Sn at high growth temperatures, it has been found that the ratio of Nb:Sn can be pinned at 3:1. The abundance of Sn drives the material into the two-phase region, where excess Sn re-evaporates from the film, avoiding the formation of Sn precipitates^23,24^. To achieve the high temperatures (> 930 °C) required for this growth, sapphire substrates were heated from behind with a SiC radiative heater. Radiation passed through the substrate and heated the depositing metal directly. Growth temperature was measured by pyrometer. Details of the film growth are given in the Supplemental Information.

A series of films was grown with fixed Nb flux (0.7 Å/s) and varying Sn flux (0.4–2.5 Å/s), and low-temperature resistance measurements were carried out to find the window for this self-regulating adsorption-controlled process. Shown in Fig. 2b are the dependencies of the critical temperature *T*_*c*_ and transition width Δ*T*_*c*_ on the deposition rate of Sn which clearly saturate at ~ 1 Å/s. Given the dependence of *T*_*c*_ on Sn content in Nb_3_Sn, this growth rate roughly corresponds to the boundary between two processing regimes. At lower flux, Sn evaporates from the film faster than it can be incorporated, resulting in a Sn-deficient film. At higher flux, sufficient Sn is provided to react with all available Nb, and only excess Sn re-evaporates.

**Figure 3:**
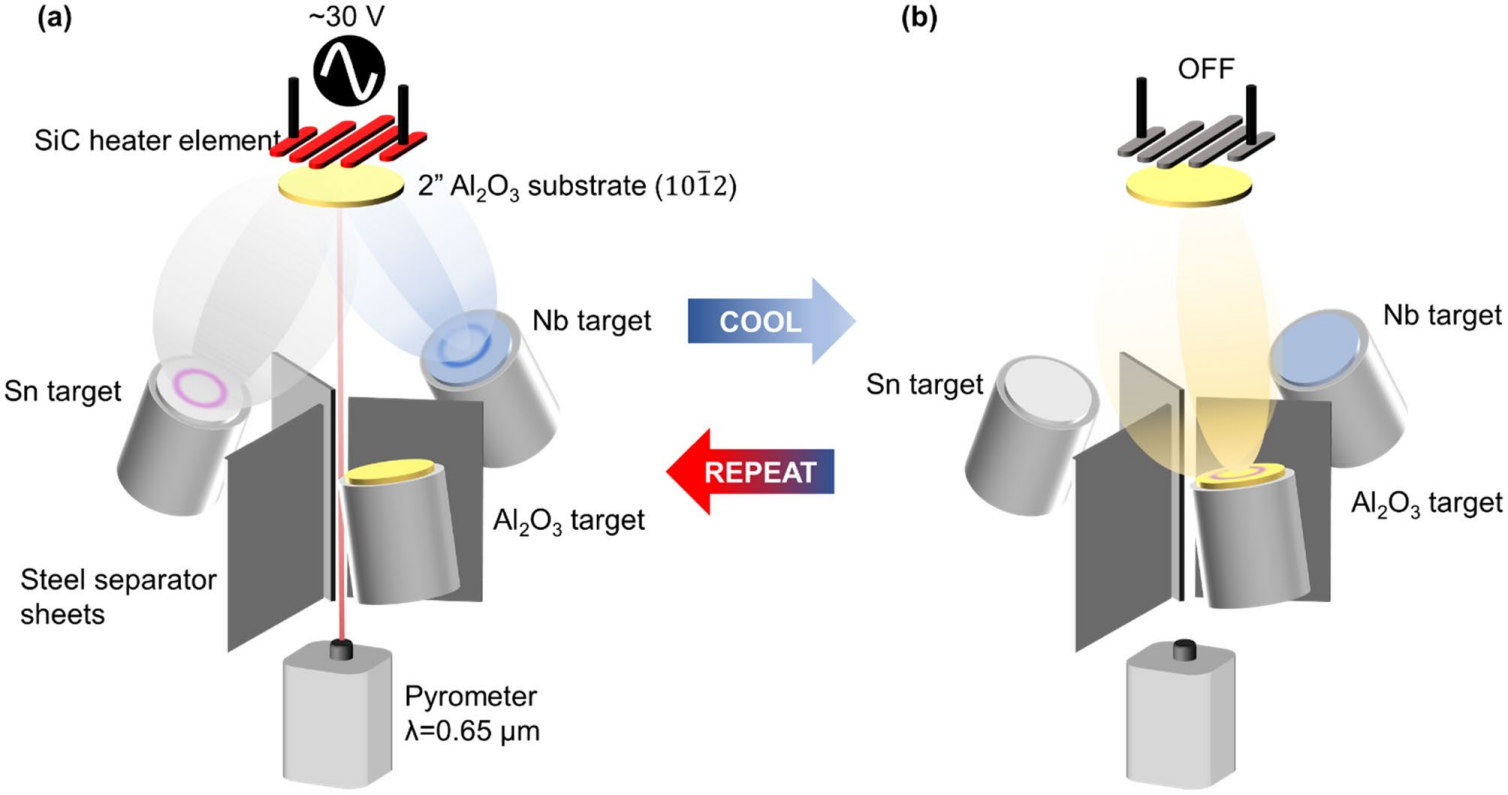
Schematic of thin films deposition setup and sequential processing steps for Nb_3_Sn/Al_2_O_3_ multilayer heterostructures. (**a**) Nb and Sn are sputtered onto Al_2_O_3_ substrate while heater element is powered on. (**b**) After allowing film to cool, Al_2_O_3_ is sputtered from a single stoichiometric Al_2_O_3_ target. Sample is heated again to anneal Al_2_O_3_. These two steps are repeated to produce multilayer samples.

The dielectric Al_2_O_3_ interlayers were grown after allowing Nb_3_Sn to cool down to < 400 °C, using a single stoichiometric target with RF power at a rate of 1.8 nm/min without any further heating applied to the substrate. Depositing under these conditions protects the SiC heater element from oxygen evolved during the sputtering process and prevents undesired reactions with the Nb_3_Sn surface. This Nb_3_Sn/Al_2_O_3_ stack was then heated again to above 900 °C, which allows the Al_2_O_3_ to crystallize, and the process was repeated to grow heterostructures of up to three Nb_3_Sn layers. The chamber setup and growth steps are depicted in Fig. 3.

### Structural characterization

**Figure 4:**
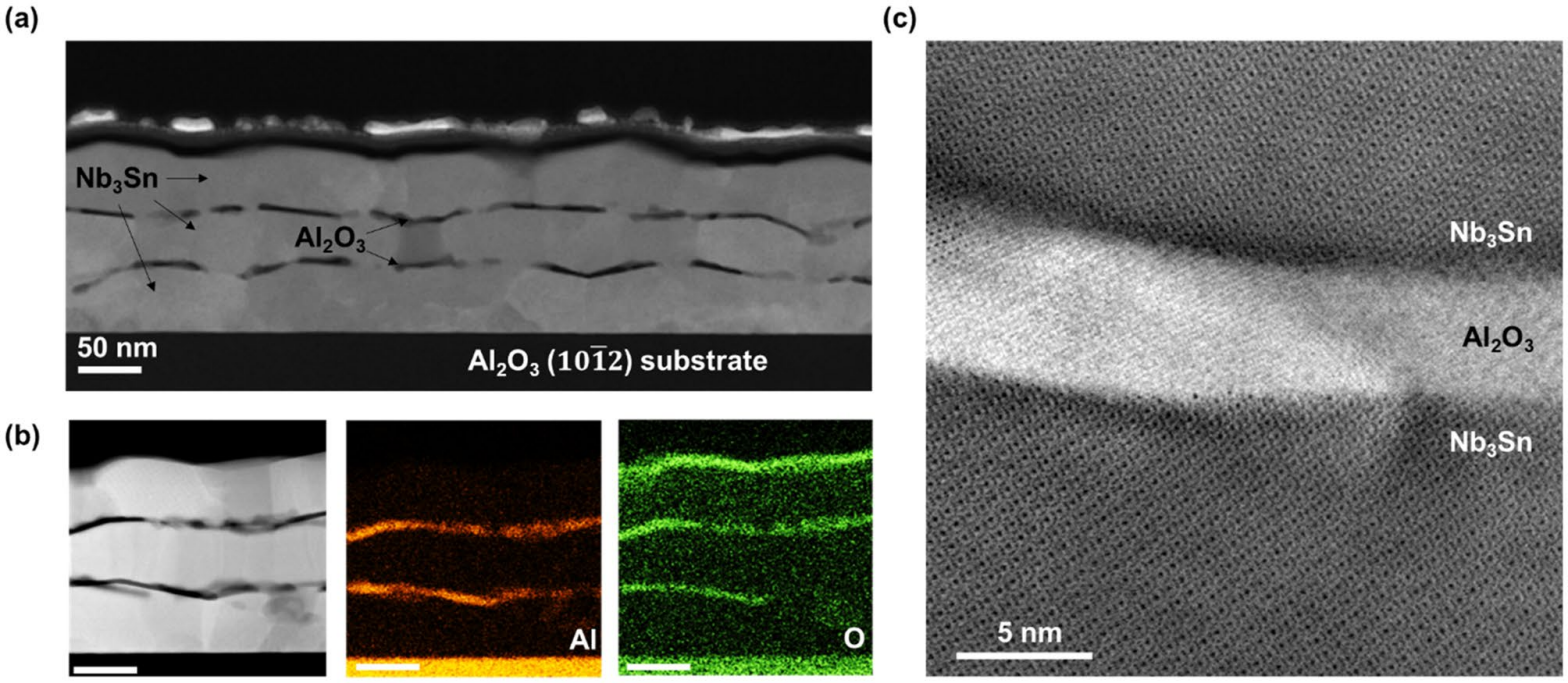
Cross-sectional transmission electron microscopy images of Nb_3_Sn/Al_2_O_3_ multilayer heterostructures (**a**) Low-magnification image of trilayer morphology. (**b**) EDS compositional mapping of Al and O showing no interdiffusion between Al_2_O_3_ and Nb_3_Sn. (**c**) High-magnification image of the interfaces between Al_2_O_3_ and Nb_3_Sn.

A SIS sample with three Nb_3_Sn layers was prepared for analysis by cross-sectional scanning transmission electron microscopy (STEM). A low-magnification image (Fig. 4a) represents the morphology and nanostructure of the stack. Each Nb_3_Sn layer is polycrystalline with irregular interfaces and grain size is 20–100 nm along the film surface direction. The Al_2_O_3_ layers conform closely to the layer below but are discontinuous along the Nb_3_Sn/Al_2_O_3_ interface. Despite the repeated thermal cycling during stacking, it appears that the lower layers have not degraded in comparison to the top layer.

The chemical stability of these films is further confirmed by compositional mapping with energy dispersive spectroscopy (EDS) (Fig. 4b). Intensity of the Al Kα and O Kα peaks are mapped from the region shown on the left. Although the Al_2_O_3_ layers are not continuous, Al and O are confined to the Al_2_O_3_ layers, and do not mix with the Nb_3_Sn layers. The exception to this is the presence of O at the interface of the topmost Nb_3_Sn film with the atmosphere, where ambient conditions are sufficient to cause a reaction. A capping Al_2_O_3_ layer could be deposited to prevent this oxidation, but we did not use it for the multilayer samples described in this work. Note that the slight O signal in the Nb_3_Sn layers in Fig. 4b are due to the slight oxidation of the TEM specimen surface. As our RF cavity measurements show, these Al_2_O_3_ layers do not contribute significantly to surface resistance at low fields.

A higher-magnification image of the S–I interface is shown in Fig. 4c. The atomic structure of Nb_3_Sn is well-preserved at the interface, suggesting that there is almost no diffusion or intermixing from the Al_2_O_3_. The lower Nb_3_Sn grain orients the [023] direction normal to the film surface, and this direction is also preserved in the upper Nb_3_Sn grain. This can occur when the upper Nb_3_Sn layer deposits with the same epitaxial relationship to the underlying Al_2_O_3_ as the lower layer has with the Al_2_O_3_ substrate. This structure can also form when a Nb_3_Sn grain nucleates on top of a Nb_3_Sn surface exposed by breaks in the discontinuous Al_2_O_3_ layer. X-ray diffractometry indicates that Nb_3_Sn grains in the second layer have more random crystallographic orientation compared to the first layer (see the Supplemental material).

### Superconducting properties

**Figure 5:**
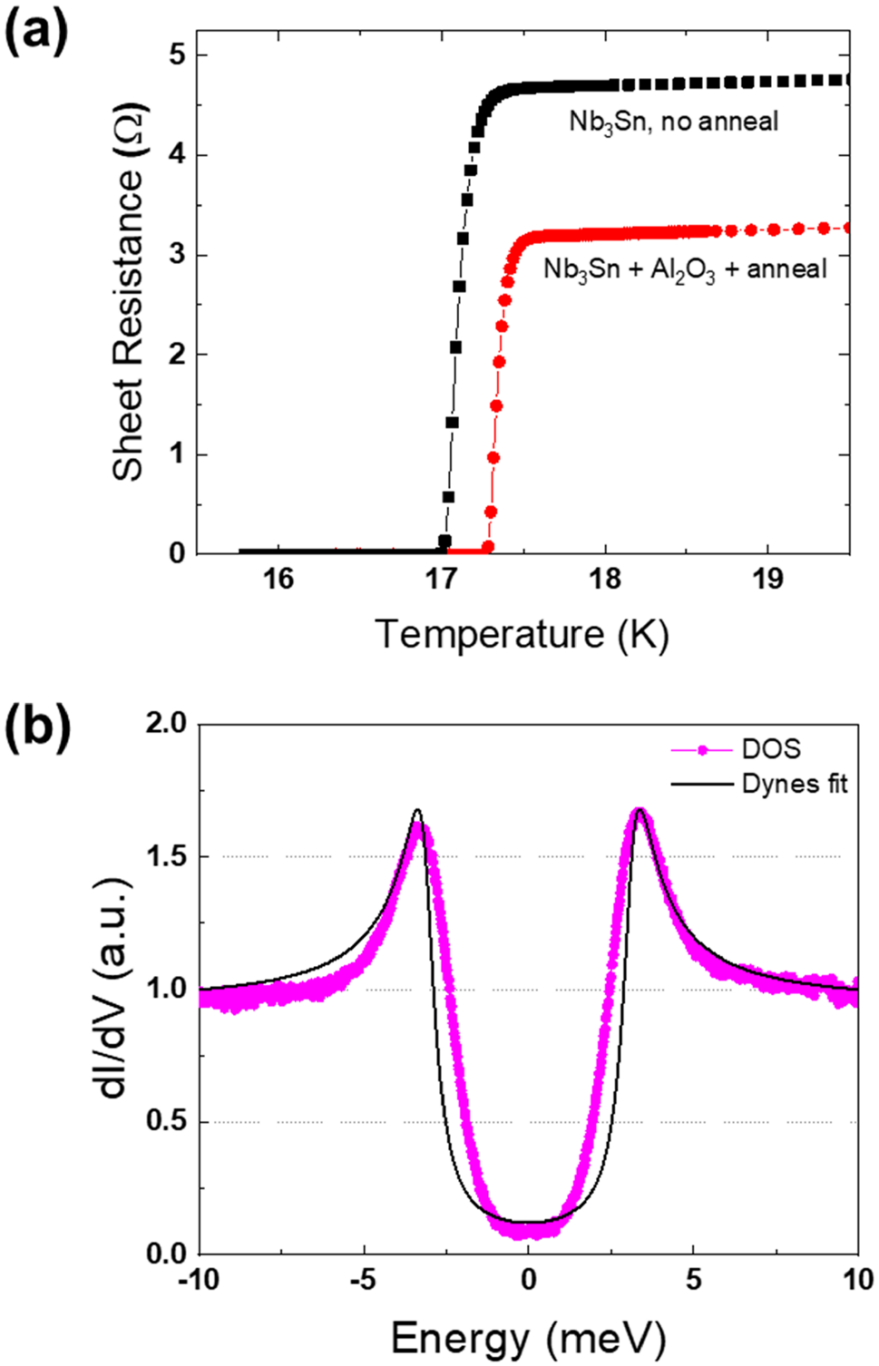
Superconducting properties of single-layer Nb_3_Sn films. (**a**) Resistive superconducting transition for two identical 60 nm thick films, one of which is capped with 6 nm Al_2_O_3_ overlayer and annealed at 900 °C for 10 min. (**b**) Density of states measured by scanning tunneling spectroscopy and Dynes fit for a 60 nm thick Nb_3_Sn film.

Our dc transport measurements have shown that the Nb_3_Sn films capped with Al_2_O_3_ and annealed with no further deposition exhibit good superconducting properties. For instance, the superconducting resistive transitions of a bare Nb_3_Sn film and a Nb_3_Sn/Al_2_O_3_ structure annealed at 900 °C for 10 min are shown in Fig. 5a. Here the critical temperature of the annealed sample is about 0.25 K higher than ${T}_{c}$ of the unannealed sample, and residual resistivity ratio (RRR), an indicator of crystalline and metallic quality, is improved from 3.5 to 4.26. On the other hand, Nb_3_Sn films annealed *without* the Al_2_O_3_ cap, even under high Sn flux to prevent evaporative loss, have degraded superconducting properties compared to an un-annealed film.

The superconducting properties essential for the RF performance were characterized by scanning tunneling spectroscopy (STS) which measures the differential tunneling conductance dI/dV proportional to the quasiparticle density of states (DOS), N(E). Shown in Fig. 5b is a representative tunneling spectrum measured in the center of a Nb_3_Sn grain at 4 K. The DOS curves, which clearly show the superconducting gap $\Delta$ at the Fermi surface, were fit using the conventional Dynes model^25,26^:

1 $$N\left(E\right)={N}_{0}Re\left[\frac{E-i\Gamma }{\sqrt{{(E-i\Gamma )}^{2}-{\Delta }^{2}}}\right]$$

where the phenomenological parameter Γ accounts for the broadening of the DOS peaks due to a finite lifetime of quasiparticles, and N_0_ is the DOS in the normal state. The fit was done with Γ = 0.4 meV and $\Delta \approx 3.1$ meV, consistent with the conventional gap value for a stoichiometric Nb_3_Sn^4^. The ratio $\Gamma /\Delta \approx 13$% in our samples turns out to be about 2–3 times larger than the values observed by tunneling spectroscopy on 1–2 μm thick Nb_3_Sn films for rf applications^27^ and Nb coupons^28^. The deviations of the STM data from the Dynes model at low energies $E<\Delta$ may indicate the effects of local non-stoichiometry, gap anisotropy and strain^22^, scattering of quasiparticles on magnetic impurities, and a thin layer with deteriorated superconducting properties at the surface^27–30^. In turn, the subgap quasiparticles states which appear at |E|< Δ due to a finite Γ contribute to a temperature-independent residual surface resistance *R*_*i*_ at *k*_*B*_*T* <  < Δ^5,29^

2 $${R}_{i}=\frac{{\mu }_{0}^{2}{\omega }^{2}{\lambda }^{3}{\Gamma }^{2}}{2{\rho }_{n}\left({\Delta }^{2}+{\Gamma }^{2}\right)}$$

Here *μ*_0_ is the permeability of free space, ${\rho }_{n}$ is the normal-state resistivity, $\lambda$ is the magnetic penetration depth, and $\omega =2\pi f$ is the circular RF frequency^5^. For *λ* = 120 nm, ${\rho }_{n}$ = 3.0 × 10^–7^ Ωm, and the fit parameters Δ = 3.1 meV and Γ = 0.4 meV, we obtain *R*_*i*_ $\approx 5.0$ nΩ at f = 1.3 GHz. This estimate is of the order of *R*_*i*_ ≈ 5–10 nΩ for large-grain Nb cavities^31^. Below a few nm thick surface layer but well within the rf penetration depth λ ≈ 120 nm, the gap peaks in the DOS are likely much sharper. There are other essential contributions to *R*_*i*_ most notably due to non-stoichiometric regions in the bulk^27^, grain boundaries and trapped vortices^32^.

### Low-field RF characterization

**Figure 6:**
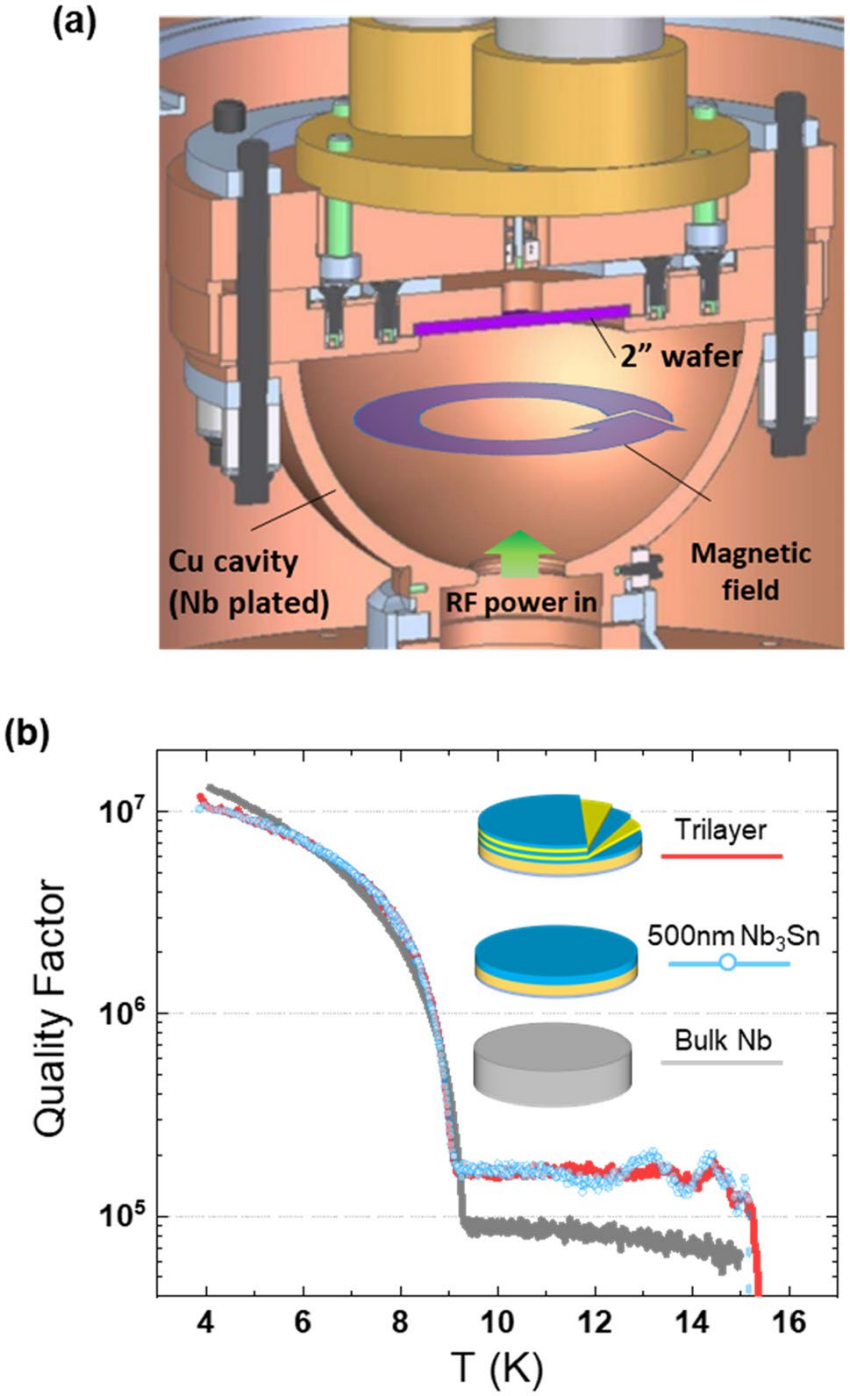
(**a**) Cutaway of hemispherical resonator cavity at SLAC used for these measurements. RF power is pumped in at the bottom, and magnetic field lines oscillate parallel to sample surface. (**b**) RF Surface resistance measurements of Nb_3_Sn film and multilayer compared to cavity-grade Nb.

Multilayer samples grown on 2″ sapphire wafers were tested in a hemispherical Nb-coated cavity at SLAC National Accelerator Laboratory. The experimental setup was described previously^33^. A rendering of this cavity is shown in Fig. 6a. The cavity operates in a TE_032_-like mode at 11.4 GHz and the surface RF field of the order of 30 μT, with a pocket on the flat face for mounting 2″-diameter samples (shown in purple). The overall cavity quality factor is measured, and the properties of the wafer can be deduced by comparison with known samples. The geometry of the cavity is engineered such that the magnetic field is strongest at the sample surface, limiting the contribution of the cavity material to the overall cavity loss. According to simulations, the participation factor is 0.33 for the 2″-diameter sample. Crucially, the magnetic field at the sample is in the radial direction and parallel to the sample surface, making it possible to measure RF properties of the sample without interference from the perpendicular component of the field. Low-field measurements of Q(T) of a Nb_3_Sn film in an uncoated Cu cavity are presented in the Supplementary Information.

The SRF performance of two Nb_3_Sn samples were compared in this system to a cavity-grade bulk Nb coupon. A 500 nm (~ 4λ) Nb_3_Sn film intended to completely screen out the RF magnetic field, and a 3 × 60 nm Nb_3_Sn/Al_2_O_3_ trilayer were tested under the RF field. Both samples were coated with a 200 nm Nb film on the backside of the wafer to prevent leakage of magnetic field as shown in Fig. 1. The quality factor of the cavity with each sample, measured at low power with a network analyzer, is plotted in Fig. 6b. The abrupt increase in Q at about 15 K corresponds to the superconducting transition of Nb_3_Sn, followed by an increase of Q(T) at *T*_*c*_ = 9 K of the Nb-coated host cavity.

As shown in Fig. 6b, the thick Nb_3_Sn film and the trilayer have nearly identical Q at T < 9 K, indicating that Al_2_O_3_ dielectric layers and interfaces do not contribute significantly to the RF dissipation. We would expect the thick Nb_3_Sn film to have a higher Q, as magnetic field is more fully screened before reaching the substrate and backside, so this result suggests that the maximum Q of these films and multilayers is limited by the quality of the Nb_3_Sn material rather than by the interfaces with Al_2_O_3_. The quality factors of both the film and the trilayer samples exceed Q(T) of Nb at T > 6 K due to the higher *T*_*c*_ of Nb_3_Sn and is about 2 times smaller than Q of Nb at 4 K.

### Discussion

The results of this work show that, despite the obvious non-stoichiometry and inhomogeneity of superconducting properties, grain boundaries, Nb inclusions, and incomplete Al_2_O_3_ layers, our multilayers exhibit the quality factors on par of those of cavity-grade bulk Nb at 4 K and low RF power. The significant local non-stoichiometry of thick (a few micron) polycrystalline Nb_3_Sn coatings of Nb cavities^20,27^, as well as Sn depletion at grain boundaries in Nb_3_Sn^34–37^ have been well documented in the literature. Yet, despite these materials issues which are also characteristic of 1–3 μm thick Nb_3_Sn films used in SRF cavities^38^, our Nb_3_Sn SIS structures exhibit higher low-field Q values than Nb at T > 6 K^20^, consistent with the larger superconducting energy gap ${\Delta }_{\mathrm{Nb}3\mathrm{Sn}}\approx 2{\Delta }_{\mathrm{Nb}}$ and a lower BCS surface resistance ${R}_{BCS}\propto {\omega }^{2}{\rho }_{n}^{1/2}{e}^{-\Delta /{k}_{B}T}$ of Nb_3_Sn. These experimental results not only show a remarkable resilience of low-field quality factors of Nb_3_Sn to the significant non-stoichiometry and materials imperfections but also suggest that the SRF performance of Nb_3_Sn coatings can be further improved by materials treatments. Our Nb_3_Sn multilayers exhibit a similar resilience of the low-power SRF performance to the materials imperfections.

The slopes of Q(T) for both the Nb_3_Sn film and multilayer shown in Fig. 5 tend to level off at 4–5 K and are clearly smaller than the slope of Q(T) for Nb. This indicates that Q(T) of the Nb_3_Sn samples at T = 4–5 K is not limited by the BCS surface resistance for which the slope of $\mathrm{Q}\left(\mathrm{T}\right)\propto {e}^{\Delta /{k}_{B}T}$ for Nb_3_Sn would be larger than for Nb because ${\Delta }_{\mathrm{Nb}3\mathrm{Sn}}\approx 2{\Delta }_{\mathrm{Nb}}$. The behavior of Q(T) of the Nb_3_Sn samples at 4–5 K is thus indicative of a significant residual surface resistance caused by the multiphase structure of the films and multilayers and trapped vortices. Yet ${\mathrm{Q}}_{0}\simeq {10}^{7}$ observed on our Nb_3_Sn multilayers at 11.4 GHz and 4 K suggests values of ${\mathrm{Q}}_{0}\sim {10}^{9}$ at 4 K and 1 GHz given the frequency dependence $\mathrm{Q}\propto {\omega }^{-2}$ which comes from the BCS surface resistance^1–3^, ohmic losses in metallic precipitates smaller than the RF skin depth and perhaps Josephson vortices trapped on grain boundaries^39^.

SRF performance at high RF fields and breakdown fields of Nb_3_Sn/Al_2_O_3_ multilayers are yet to be explored. Generally, the effects of nonstoichiometry, proximity-coupled normal precipitates and weakly-coupled grain boundaries become more pronounced at higher RF fields. For instance, nonstoichiometric grain boundaries in Nb_3_Sn have been identified as prime pinning centers for vortices in Nb_3_Sn wires for high-field dc magnets^39^. However, weakly-coupled grain boundaries in Nb_3_Sn coating layers would block RF currents and cause dissipative penetration of Josephson vortices at fields well below the superheating field^40^, and sub-stoichiometric regions in Nb_3_Sn-coated Nb cavities are suspected to play an important role in RF cavity quench^27^. At the same time, meandering and breaks in Al_2_O_3_ layers shown in Fig. 4 may not be detrimental for SRF performance as the layers can still provide their main role of intercepting and pinning small vortex loops originating at surface structural defects^5,8^ since the pinholes sizes 10–50 nm in the Al_2_O_3_ layers are smaller than magnetic size of the vortex $\lambda \simeq 100{-}200$ nm of Nb_3_Sn. The misaligned breaks with lateral sizes smaller than the Nb_3_Sn layer thickness in neighboring dielectric Al_2_O_3_ layers are not expected to strongly deteriorate the SRF performance of multilayers. Such imperfect dielectric layers still produce effective pinning barriers against penetration of parallel vortices and arresting vortex semi-loops originating on surface materials defects, which is instrumental in the multilayer approach^5,6^. At the same time, the roughness and breaks in Al_2_O_3_ interlayers, as well as the variable thickness of Nb_3_Sn layers can pin short perpendicular vortices and do not let them propagate along the layers under RF current, which is also beneficial for the SRF performance of multilayers^5^. Though Al_2_O_3_ layers do not fully separate Nb_3_Sn layers, we found that a 500 nm thick Nb_3_Sn film had a quality factor identical to a multilayer with three 60 nm Nb_3_Sn layers separated by 6 nm Al_2_O_3_, and both had Q approximately 2 times lower than a cavity-grade Nb reference. This indicates that losses in the thin Al_2_O_3_ and the oxide-metallic interfaces do not contribute much to the surface resistance of our multilayer samples.

## Conclusions

In summary, we have developed a self-regulating, adsorption-controlled process for growth of Nb_3_Sn films and Nb_3_Sn/Al_2_O_3_ multilayers. We have produced and characterized multiple multilayer samples with up to four superconducting layers. Despite the detrimental effects of nonstoichiometry, grain boundaries and breaks in the meandering Al_2_O_3_ interlayers, the SRF performance of our multilayers turned out to be on par with that of Nb films. The growth technique reported in this work provides a platform for further optimizations of the SRF properties of SIS high-performance multilayers for superconducting resonator applications.

## Methods

Film deposition was carried out in a vacuum chamber pumped down to 3.0 × 10^–8^ Torr before being backfilled with Ar. Nb_3_Sn films were sputtered from elemental Nb (99.95%) and Sn (99.99%) targets in 3 mTorr of Ar to maximize deposition rate, at a distance of 15.5 cm from the substrate to improve flux uniformity. DC power to the sputter guns was current-controlled, and deposition rate was measured with an in situ quartz crystal monitor prior to growth. Pyrometer reading of the SiC heating element at the beginning of growth was ~ 1250 °C, and dropped to around 905 °C after 60 nm was deposited. Al_2_O_3_ was sputtered from a stoichiometric 2″ diameter ceramic target after the pyrometer reading fell below 400 °C. After deposition, the temperature was ramped back up to a pyrometer reading of 905 °C over the course of 10 min. These two steps were repeated to produce the multilayers.

Scanning transmission electron microscope (STEM) imaging and elemental analysis were performed in a probe-corrected atomic resolution analytical electron microscope (JEM-ARM200cF, JEOL) with an energy dispersive X-ray spectroscopy (EDS) detector (X-Max^N^ 100TLE SDD, Oxford Instruments).

Superconducting transitions were measured in a closed-loop He cooler using 4-point van der Pauw geometry on 10 × 10 mm samples. The critical temperature *T*_*c*_ is defined as the temperature at which the sheet resistance falls below 1% of its normal state value at 18 K. The transition width Δ*T*_*c*_ is defined as a difference between *T*_*c*_ and the point at which the lines drawn through the normal-state resistance and transition region intersect.

Low-temperature scanning tunneling microscopy/spectroscopy (STM/S) measurements were carried out in a STM system (USM1300, UNISOKU) at 4 K using polycrystalline PtIr tips. The dI/dV spectra were acquired using standard lock-in technique by applying a bias modulation of 0.2 mV (r.m.s.) at 732 Hz.

## Supplementary Information

Supplementary Information.

## Data Availability

The data that supports the findings of the work are in the manuscripts main text and Supplementary Information. Additional data are available from the corresponding author upon reasonable request.
