# Supplementary material for: Development and characterization of Nb3Sn/Al2O3 superconducting multilayers for particle accelerators
Source: Sci Rep. 2021 Apr 8;11:7770. doi: 10.1038/s41598-021-87119-9 (PMC8032729; doi:10.1038/s41598-021-87119-9)
Supplement: Supplementary file 1 — Supplementary Information. [file 41598_2021_87119_MOESM1_ESM.pdf]

Supplemental Information

Development and characterization of Nb_3Sn/Al_2O_3 superconducting multilayers for particle accelerators

Chris Sundahl[1], Junki Makita[2], Paul B. Welander[3], Yi-Feng Su[4], Fumitake Kametani[4,5], Lin Xie[6], Huimin Zhang[7], Lian Li[7], Alex Gurevich[2]*, Chang-Beom Eom[1]*

[1]Department of Materials Science and Engineering, University of Wisconsin-Madison, Madison, WI 53706.

[2]Physics Department and Center for Accelerator Science, Old Dominion University, Norfolk, VA 23529.

[3]SLAC National Accelerator Laboratory, Menlo Park, CA 94025.

[4]Applied Superconductivity Center, National High Magnetic Field Laboratory, Florida State University.

[5]Department of Mechanical Engineering, FAMU-FSU College of Engineering, Tallahassee, FL 32310.

[6]Department of Physics, Southern University of Science and Technology, Shenzhen, 518055, China.

[7]Department of Physics and Astronomy, West Virginia University, Morgantown, WV 26506.

*eom@engr.wisc.edu, agurevic@odu.edu

Temperature monitoring in radiative heating system

Prior to 2” wafers, growths were optimized on 10 x10 mm square substrates. A 2” diameter molybdenum plate was mounted into the heater in place of the wafer. A hole was cut through the Mo substrate block and the substrate mounted inside of it, as shown in Figure S1.

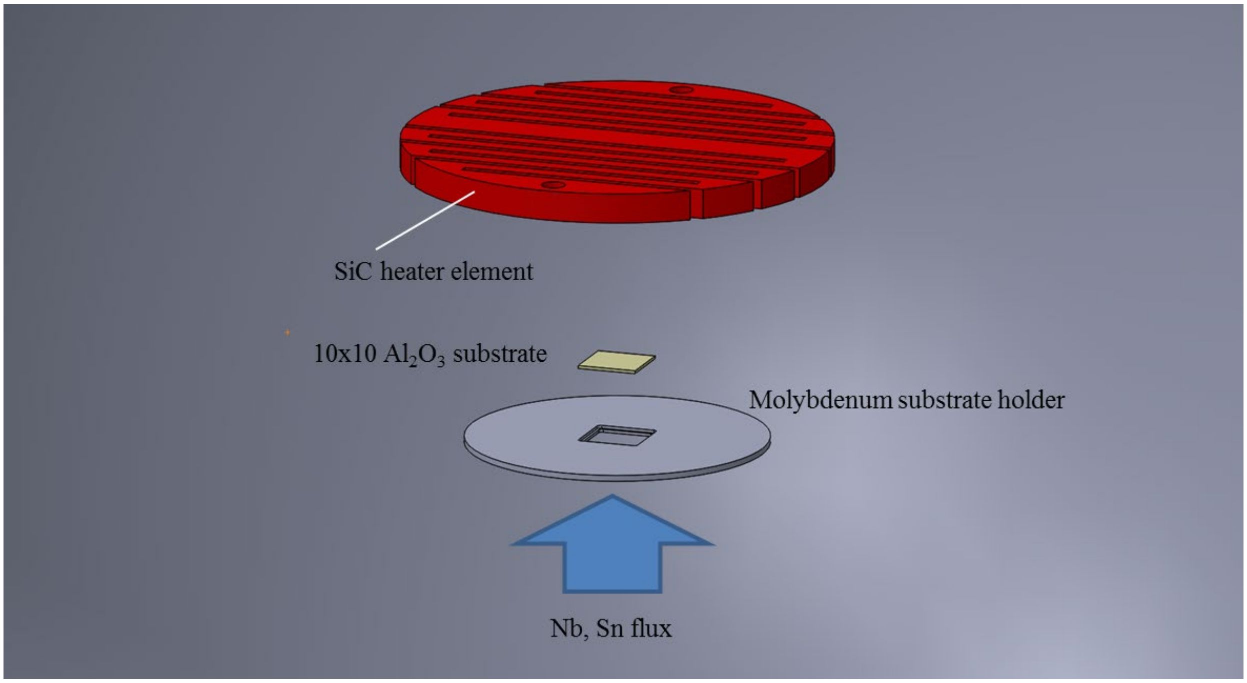

Figure S1: Close-up schematic of the heater configuration. Substrate is exposed to heater radiation from above, while Nb and Sn is sputtered from below through a hole in the Mo substrate holder.

The temperature during the growth was measured by both optical pyrometer and thermocouple. An optical pyrometer was pointed at the substrate, mounted to the bottom of the deposition chamber and peering through a CaF_2 window. Since Al_2O_3 is transparent to infrared rays, this effectively monitored the temperature of the heating element during growth, until the Nb_3Sn film grew thick enough to obscure the element from view. The value of the emissivity was chosen to be $\varepsilon = 0.35$, based on emissivity data on Nb from [1,2]. Though this likely does not match the true emissivity of Nb_3Sn, the value of monitoring with the pyrometer is in being able to compare the behavior of the pyrometer reading (T_p) between samples. With this goal, the emissivity was chosen and maintained between all samples.

This provided more consistent temperature information, but the behavior of the pyrometer reading T_p proved to be complex over the course of growth.

Figure **S2**a shows T_p over the course of a growth on a 10x10 mm substrate. Only the heater voltage was regulated for this growth. Rotation of the heater starts at the point labeled as 1, where oscillations appear in T_p. This is due to the rotation causing the heater element to travel across the pyrometer's viewing window, changing the observed temperature, as illustrated in

Figure **S2**b. Sputter guns are switched on at 2, and T_p immediately begins to drop as the film begins to obscure the heater element. Oscillations from rotation begin to dampen as the film becomes thicker, and at point 3 the oscillations have dampened considerably and T_p approaches a constant value. Power to the heater is switched off at point 4, and T_p declines rapidly.

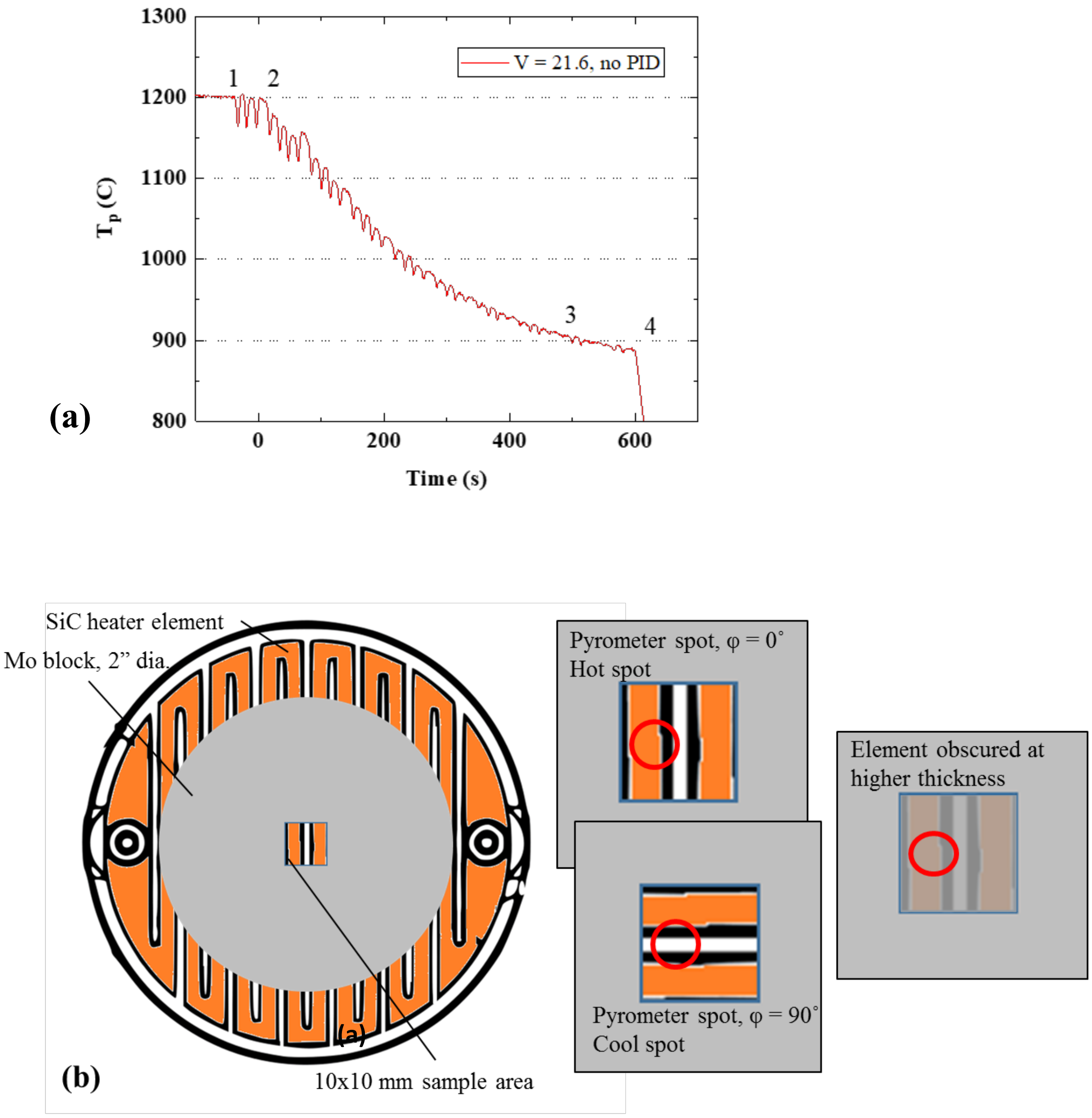

Figure S2: (a) the behavior of T_p during film deposition. (b) Schematic of the heater/sample configuration, visualizing the cause of oscillations in T_p and damping of oscillations during film growth.

It is difficult to reproduce this behavior from one film growth to the next, especially when the films can vary in composition. Changes in composition can contribute to small differences in emissivity, resulting in inconsistent T_p readings from which it is difficult to discern what conditions have been

kept constant. When growing films that are substantially thinner than the one in Figure **S2**, temperature does not saturate, leading to even higher uncertainty for thin films.

A hybrid method was adopted as a solution. The optical pyrometer was pointed at the substrate. In parallel, a small 0.5 mm diameter K-type thermocouple was inserted into the Mo substrate holder to monitor the temperature of the holder itself. Though the holder is not tightly thermally coupled to the substrate, this is a way to monitor the total thermal energy flowing from the heating element. The reading from the thermocouple was fed into a PID temperature controller, which manipulated the duty cycle of the heater through a relay to maintain the molybdenum substrate holder temperature (T_{Mo}) at a constant value. This setup is shown in Figure S3.

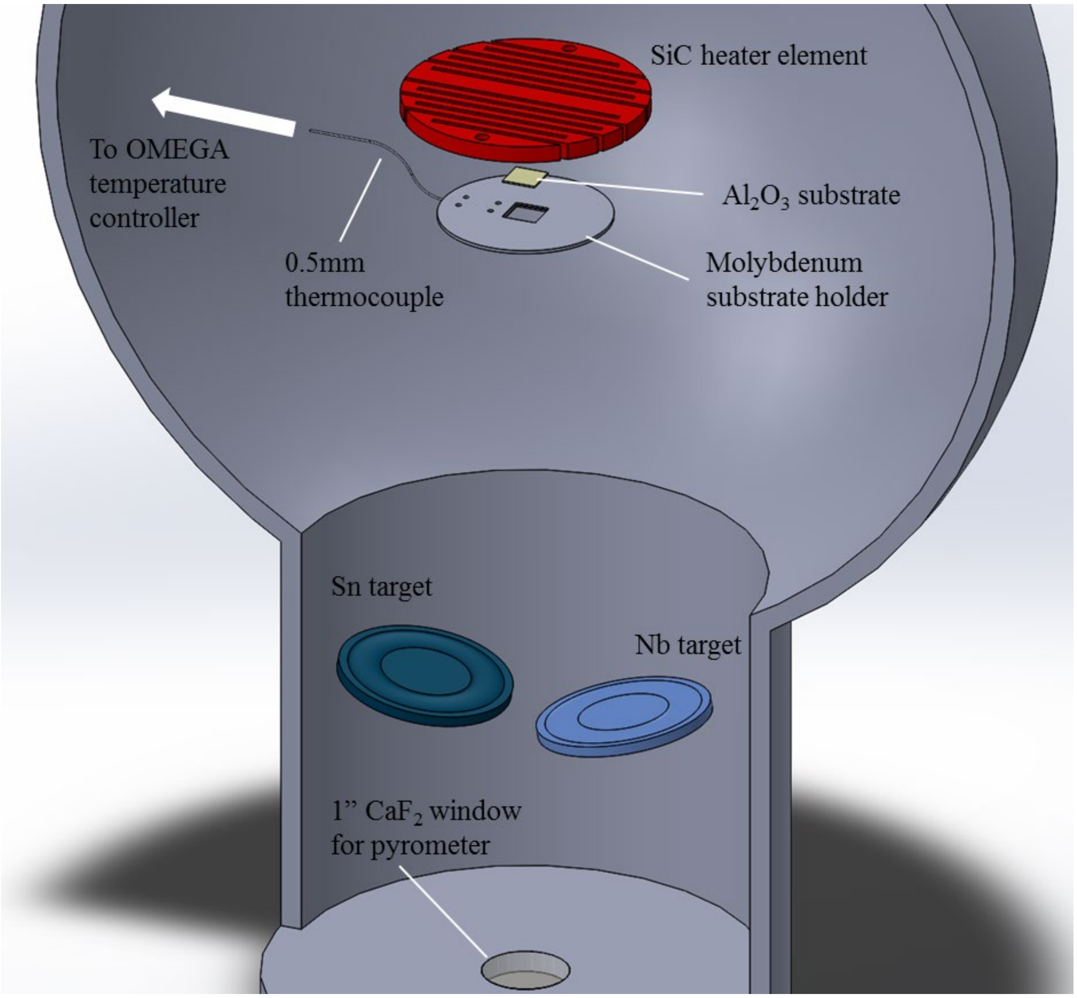

Figure S3: Chamber setup for monitoring both T_{Mo} and T_p.

With all other conditions fixed, Nb_3Sn films were grown at T_{Mo} = 800, 825, and 850 °C; this resulted in the T_p curves shown in Figure S4a. T_p curves follow the T_{Mo} setpoint, resulting in a final T_p at the end of 10-minute growth plotted in Figure S4b. This demonstrates that this method allows a degree of control over the heat transmitted to the film, even though T_{Mo} is not a direct reading of the substrate temperature.

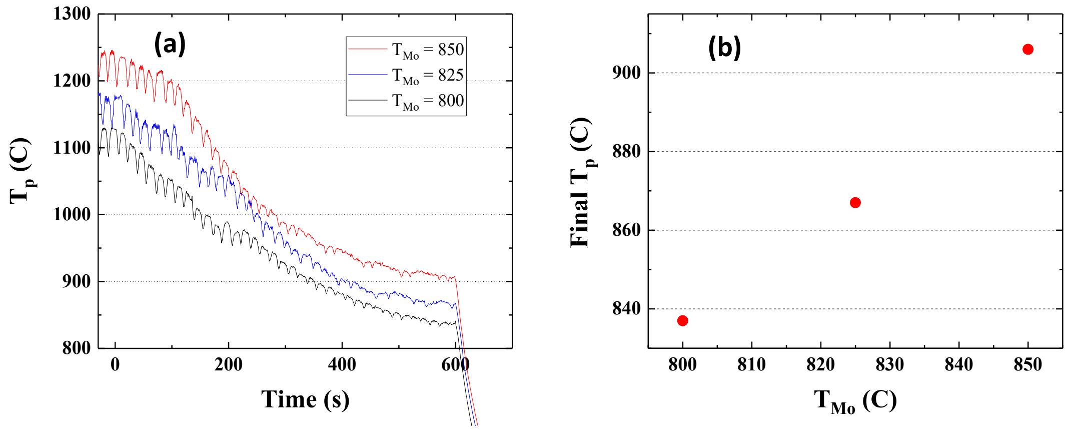

Figure S4: (a) T_{p} behavior for various setpoints of T_{Mo}. The ending T_{p} is plotted in (b), and appears linear with T_{Mo}.

When growing 2” wafers, this method presents some challenges. The wafer is situated where the Mo substrate holder would typically be. Because the holder is no longer part of the system, there is no comparable place to situate the thermocouple; these films are grown with only a T_{p} reading as a way of regulating growth temperature. Reference data from a film grown on a 10x10 mm substrate with the same Sn flux was used to match T_{p} during wafer-scale growth.

DC Characterization of wafer cutouts

A 60-nm thick film was grown on a 2” Al_2O_3 wafer and diced for comparison to 10x10 mm samples. Sections of this wafer from the center and edges were cut out, as shown in Figure S5. Cutout 2, from the edge nearest the current lead, is where the growth temperature is expected to be lowest. T_c is not significantly different far from the center (17.2 vs 17.07 K), although the transition width seems to be affected (0.13 K at the center vs. 0.58 K at the edge); there is a variation in sheet resistance, which may be due to film thickness variation (film is thinner at the edges). RRR ranges from 3.23 to 3.71 over the three cutouts, confirming that the quality of the film does not vary significantly over the wafer area.

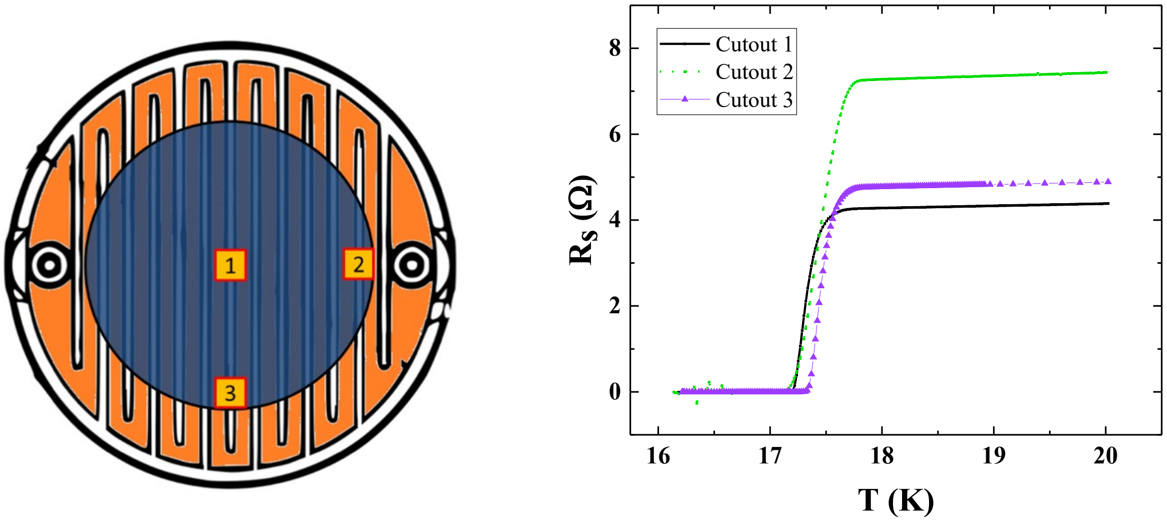

Figure S5: Superconducting transitions of pieces cut from a 2" wafer. Variation in T_c is <2%.

Contrary to expectation, T_c at the edge in Cutout 3 was higher than the center (Cutout 1) by ~0.1 K. All regions at the edge of the wafer should receive equal Sn flux, since the heater rotates at a rate of ~2 rpm, which means this cutout is only differentiated from Cutout 2 by the heater flux it received. The combination of heater flux, Nb/Sn ratio, and total growth rate at Cutout 3 seems to be optimal for producing higher T_c in these films, though the small variations in superconducting parameters should have minimal effect on the RF properties of the wafer.

Epitaxial relationship of Nb_3Sn with Al_2O_3 and $LaAlO_3$ substrates

In the early stages of this project, the goal was to grow epitaxial thin films of Nb_3Sn on low-loss dielectric substrates. We experimented with several substrates and orientations in pursuing this goal. All of the films in this section on substrate orientation were grown at the same conditions, with T_{Mo} = 870 ˚C, Sn flux (r_{Sn}) = 1.5 Å/s, and Nb flux (r_{Nb}) = 0.7 Å/s.

R-plane $(10\bar{1}2)$ Al_2O_3

These films are oriented with the (023) planes parallel to the substrate. XRD patterns on a 2-dimensional detector are shown in Figure S6a. The (012) reflections are present on either side of the q_{normal} line with equivalent intensity, revealing that two distinct in-plane orientations of (023) grains are present, with the [100] film direction oriented along the $[\bar{1}011]$ substrate direction. Equivalent intensity in (012) and $(0\bar{1}2)$ indicate that there is no preference in the system between these two directions. Figure S6b and Figure S6c show schematics this epitaxial relationship from two different perspectives.

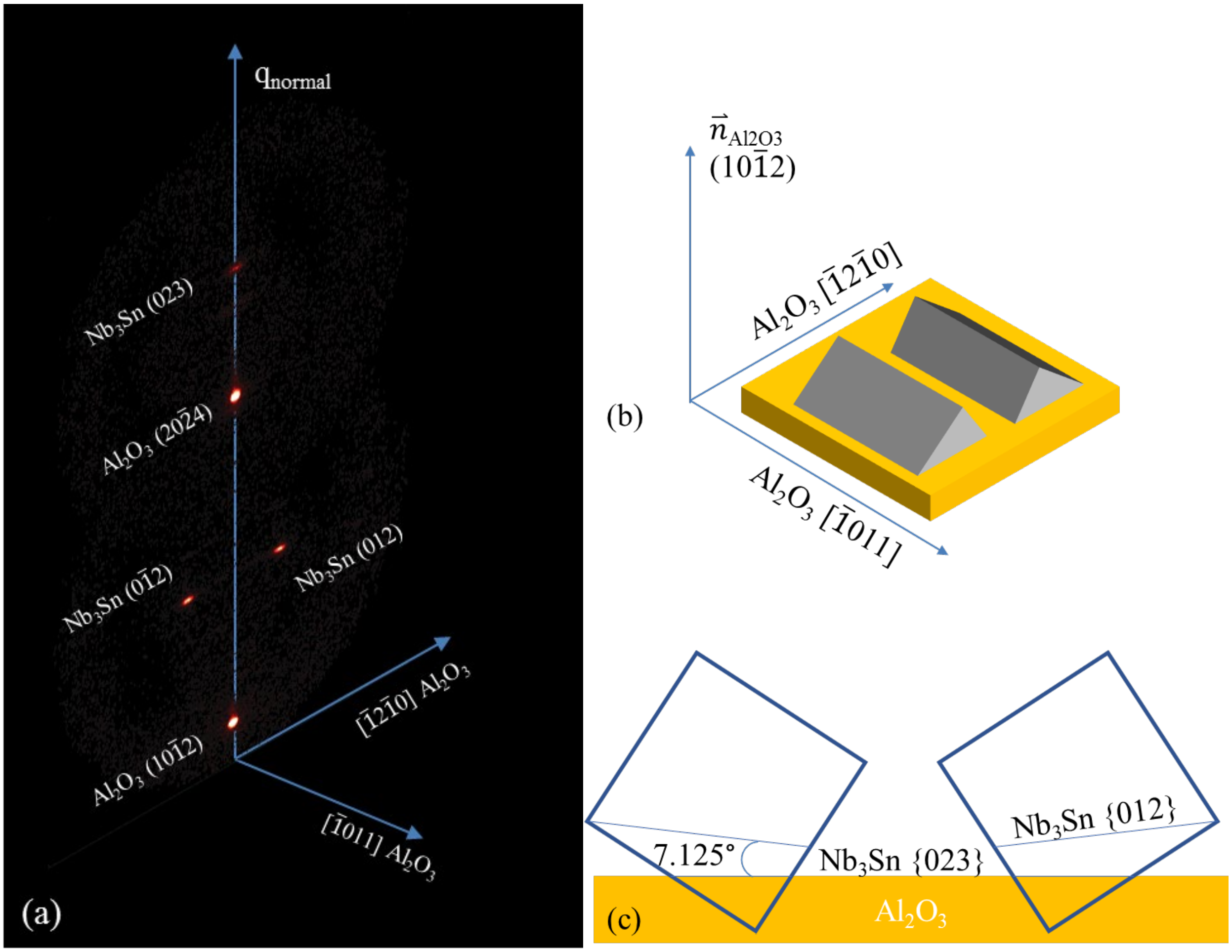

Figure S6: (a) 2D-XRD data on Nb_3Sn/R-Al_2O_3, visualized as a reciprocal space map. Nb_3Sn (012) peak is split, with each peak at $\chi = \pm 7.1$ degrees from q_{normal}. (b) 3D schematic of the epitaxial relationship between Al_2O_3 and Nb_3Sn in this system. Both possible grain orientations are represented. (c) 2D schematic, with blue boxes indicating the cubic unit cell of Nb_3Sn.

AFM images reveal that the grains are ridged, with the long axis of the grains aligned along the [100] direction of the Nb_3Sn, as shown in Figure S7. Sections of these images show that the faces of the grains are angled at ~7° relative to the substrate. This suggests that the faces are (012) planes, which are at an angle of 7.125° to the (023) plane around the [100] zone axis.

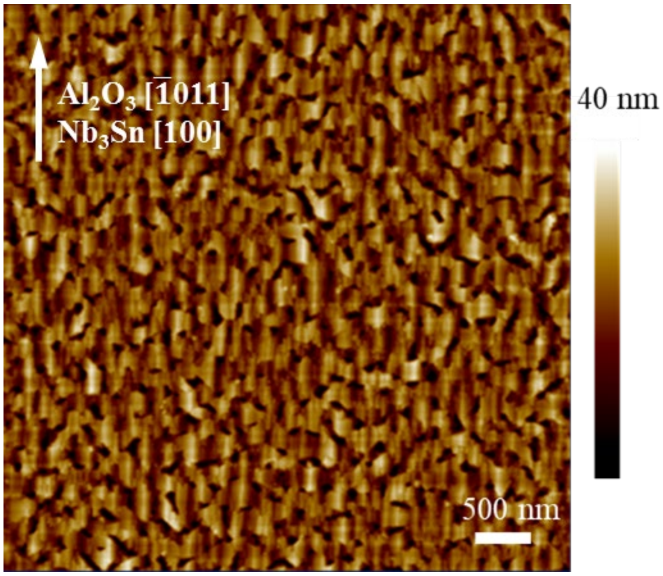

Figure S7: AFM image of Nb_3Sn thin film on R-plane Al_2O_3 substrates Ridged grains are aligned with [-1011] direction of Al_2O_3.

This is consistent with literature information on the R-plane surface of Al_2O_3 [3,4], visualized in Figure S8. The surface of the R-plane is composed of rectangular cells with periodicity of 4.76 Å in the $[\bar{1}2\bar{1}0]$ direction and 5.13 Å in the $[\bar{1}011]$ direction. The (001) spacing of Nb_3Sn is 5.29 Å, matching with the $[\bar{1}011]$ spacing of Al_2O_3 with a 3% mismatch; this relationship of [100] Nb_3Sn to $[\bar{1}011]$ Al_2O_3 matches what we see in the XRD and AFM data. The periodicity in the [032] direction of Nb_3Sn is 4.77 Å, leading to a remarkably small lattice mismatch of 0.3% in this orientation.

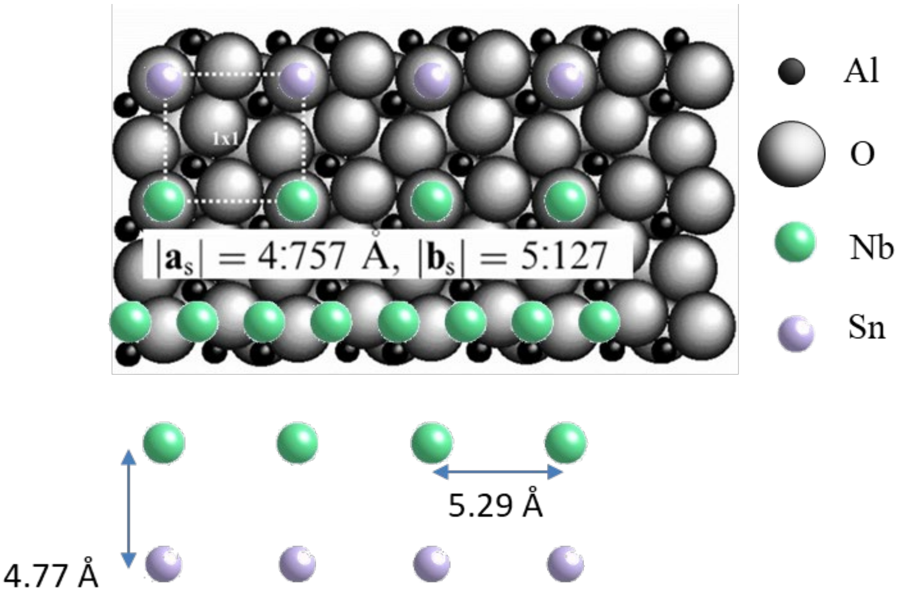

Figure S8: R-plane surface of Al_2O_3 and epitaxial relationship to Nb_3Sn (023). Al ions are represented in black, and O ions are light; overlaid Nb atoms are green, and Sn atoms are purple. Surface unit cell and lattice parameters are indicated. Figure adapted from [4].

This ridged structure of the grains leads to a high surface roughness, depending on the size of the grains, making true thickness of the film difficult to discern. Depending on growth conditions, RMS roughness could range from 3.5 to 26 nm for superconducting films. For SRF and SIS applications, rougher films are not ideal, especially when the thickness of the film is a critical parameter in determining the vortex penetration field of the structure.

A-plane (11$\bar{2}$0) Al_2O_3

We also grew Nb_3Sn films on (11$\bar{2}$0) oriented Al_2O_3 substrates at the same conditions. This also led to textured films, with the (012) orientation parallel to the surface. This still leaves two possible grain orientations; the X-ray scans in Figure S9 show that the (023) peak has split into two, at a 7.1° angle from the substrate normal. This is the reverse of the situation found in R-plane Al_2O_3. Since the brightest film peak lies in the same plane as the substrate, this orientation lends itself to point-detector scans much more easily.

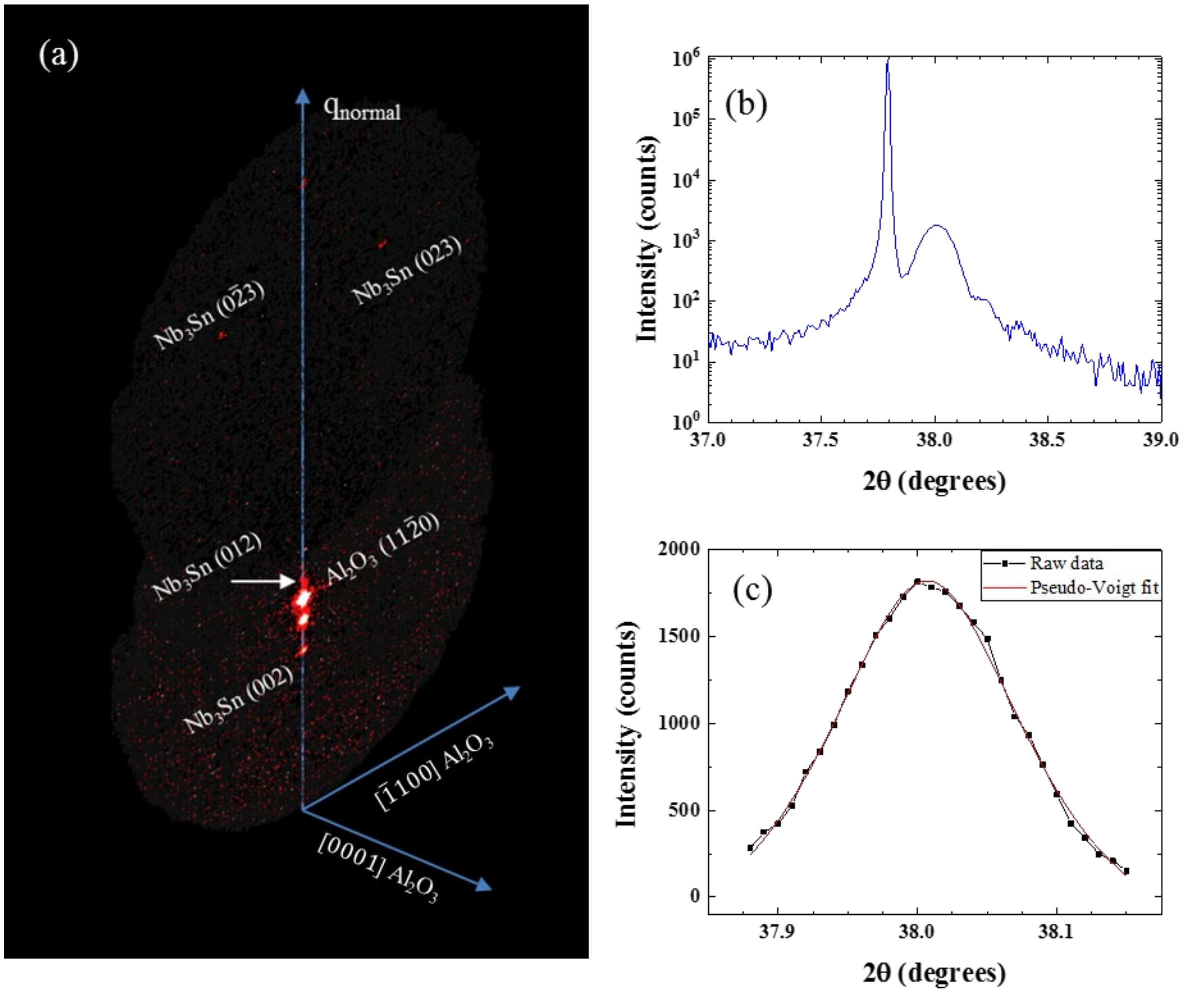

Figure S9: (a) 2D-XRD of Nb_3Sn on A-plane Al_2O_3. (012) plane is now a single point, and the (023) peak has split in turn. (b) point-detector scan of this sample around the (012) peak of Nb_3Sn, which is fit with a pseudo-Voigt peak function in (c).

Figure S9b shows such a scan, with the film peak immediately next to the substrate peak. The film peak is at $2\theta = 38.007$ °, which gives a plane spacing of 2.366 Å, or a lattice parameter of 5.2896 Å. The literature value of the bulk lattice parameter is 5.29 Å at 25% Sn, and decreases continuously across the single-phase region to 5.281 Å at 18% [5–7]. This indicates that, if strain plays a minimal role in shifting this peak position, these films are very close to stoichiometric.

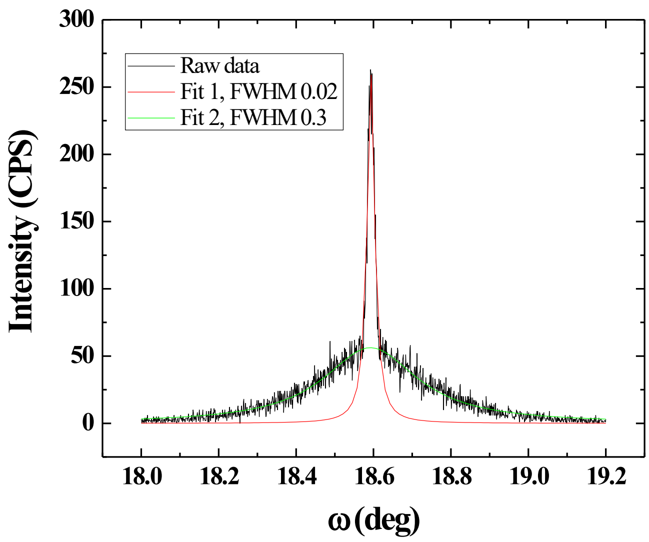

Figure S10: Rocking curve of the (012) peak of Nb_3Sn on A-plane Al_2O_3. It was fitted with two Gaussian peak functions, showing two clear contributions.

A rocking curve of the film peak (Figure S10) shows two contributions: a broad, low peak, fitted with a FWHM of 0.3 °, and a sharp peak with a FWHM of 0.024 °. This suggests that there is a strained layer at the interface between the Al_2O_3 and Nb_3Sn, and the film relaxes to the bulk value above this layer.

LAO (001)

$LaAlO_3$ (LAO) was the only cubic substrate we tested with these films. The (100) plane spacing of Nb_3Sn is roughly twice that of the (110) of LAO, making it a good candidate for (001)-oriented epitaxy of Nb_3Sn on an oxide. A film without grain boundaries and chasms would be ideal for studying the intrinsic properties of Nb_3Sn unhindered by the coupling and scattering that happens across grains; however, LAO has high dielectric losses compared to Al_2O_3, so it is unlikely to be used for SRF purposes. As such, it was only investigated here as a way to explore new possibilities for studying Nb_3Sn films.

These films are also highly textured, but are dominated by (001) oriented grains, as reflected in the 2D and 1D x-ray scans (
Figure S11). (012) grains are also present, though at a lower intensity than the (001) grains, and a faint trace of (112) reflections are visible. These results suggest that, with tighter control, purely

(001) single-crystal films could be achieved, in which intrinsic properties could be studied. Given the constraints of this project, however, this was not pursued further.

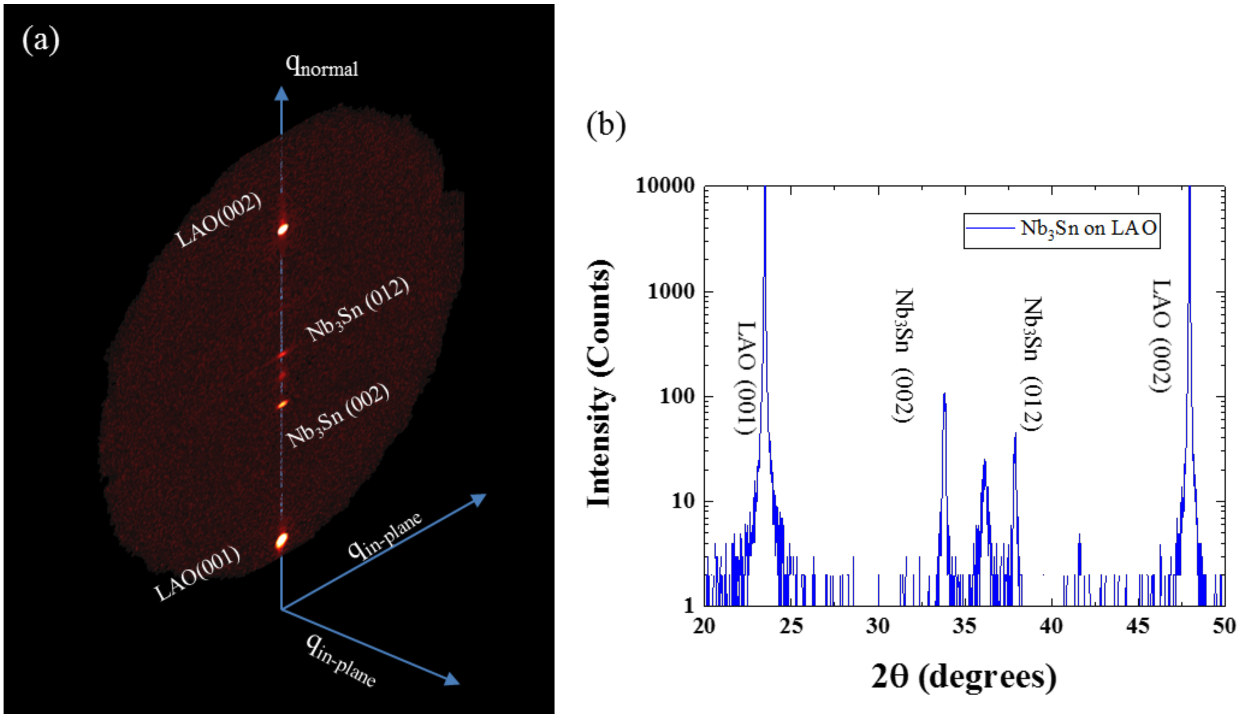

Figure S11: (a) 2D-XRD of Nb_3Sn on LAO(001). Nb_3Sn (002) is the strongest peak, but (012) is also present, along with a peak between the two that does not belong to either LAO or Nb_3Sn. (b) Point-detector scan of the same sample.

Parasitic phase

The peak that appears between the Nb_3Sn (002) and (012) peaks does not belong to either LAO or Nb_3Sn, and appears in films grown on A-plane Al_2O_3 as well. Figure S12a superimposes the XRD scans of films grown on these two substrates, showing that the peaks align, and are likely the same phase. One possibility is that O is diffusing into the Nb from the substrate, forming Nb_2O_5.

However, when Nb_3Sn is grown on CaF_2 substrates, this peak is still present (Figure S12a,b), ruling out the possibility of O supply from the substrate. This indicates that it is more likely that the parasitic phase is growing with the film, incorporating only Nb and Sn. $NbSn_2$ is a possible candidate.

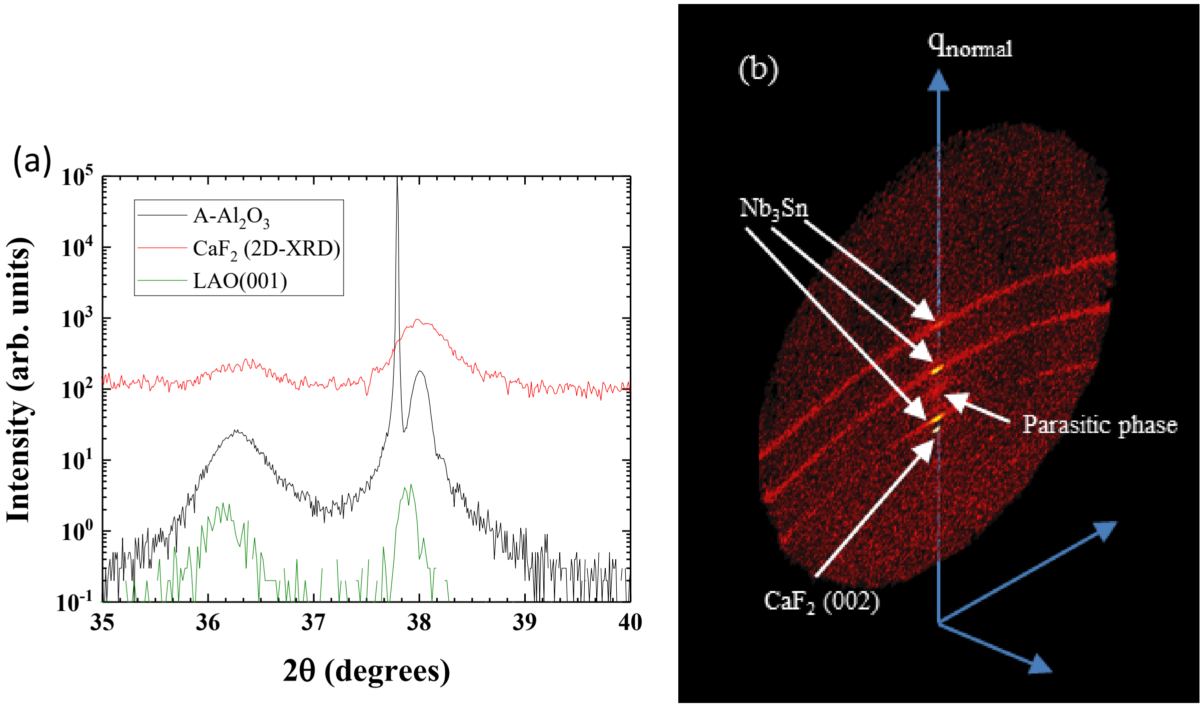

Figure S12: (a) XRD patterns of Nb_3Sn grown on A-Al_2O_3, LAO(001), and CaF_2(001), all of which show the parasitic phase peak. (b) 2D-XRD of Nb_3Sn on CaF_2(001). Parasitic phase between Nb_3Sn (002) and (012) is still present, ruling out the possibility that oxygen diffuses from the substrate into the film during growth to form the parasitic phase.

However, when Nb_3Sn is grown on R-plane Al_2O_3, parasitic peaks do not appear, as evidenced in X-ray scans in Figure S6. It is possible that the grains of this phase choose a different direction to point in, and simply do not appear in the 2D-XRD images. With this information, films were optimized and measured on R-plane substrates.

Surface and crystallographic characterization of SIS multilayer

A multilayer with two 60 nm Nb_3Sn films sandwiching a 6 nm Al_2O_3 layer was grown to characterize the surface and crystallographic structure of the upper layer. AFM image of the sample (Figure S13a) shows that much of the (023) orientation of the first layer transfers into the top layer. However, other orientations are present at a larger proportion than in bare Nb_3Sn. XRD data confirms this. Figure S13b shows that the (012) peaks are still present and distinct, but polycrystalline rings are superimposed over these peaks. This suggests that the epitaxial relationship between the Al_2O_3 film layer and the first Nb_3Sn layer is complex, and the crystal directions are not maintained between the two Nb_3Sn layers.

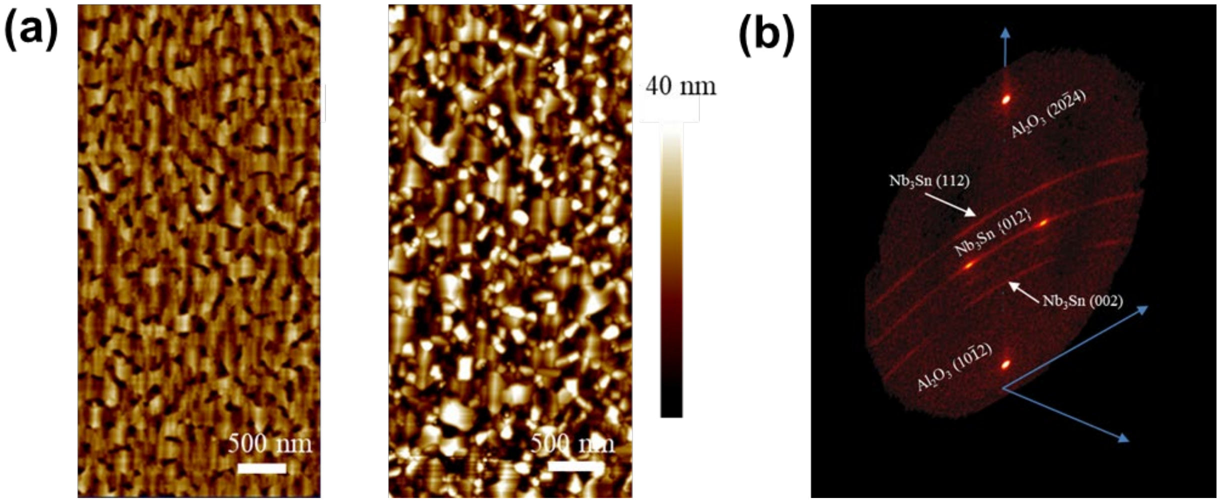

Figure S13: (a) AFM images of a single 60 nm Nb3Sn film (left) and a SIS multilayer (right). (023) orientation is cleary preserved on the right, but many other crystal orientations are also present. 2D XRD scan of SIS sample (b) shows (012) peaks from first layer, as well as polycrystalline rings.

Nb presence

Composition mapping by EDS (Figure S14) indicates that there are regions with significant Sn deficiency in the Nb_3Sn layers. High-magnification images show two phases at the interface of the Al_2O_3 substrate and Nb_3Sn film. Two grains are present: on the left, the A15 structure of Nb_3Sn is viewed along the (001) zone axis, with the [023] direction pointing up. On the right, a different orientation is present. This grain corresponds to a BCC structure viewed along the (110) zone axis, with the [001] direction pointing up.

Nb is a BCC material, and the only stable phase in the Nb-rich portion of the Nb-Sn system is pure Nb. These results strongly suggest that elemental Nb grains exist in these films inside of the Nb_3Sn matrix. In high-field environments, these grains could be weak points where magnetic field penetrates and causes quench at lower fields than expected.

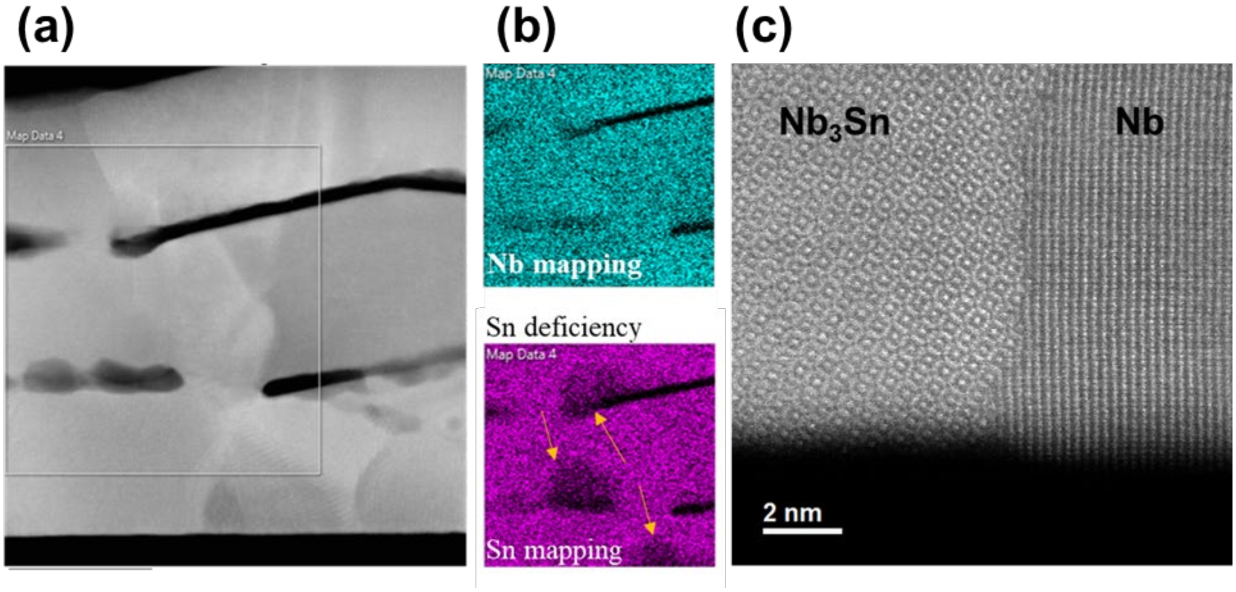

Figure S14: Cross-sectional TEM EDS compositional mapping of a trilayer. The region from (a) is mapped for Nb and Sn in (b), where Sn-deficient are evident. These do not correspond to Al_2O_3 layers. The two grains in the high-magnification image in (c) match the [100] axis of Nb_3Sn and the [110] axis of Nb, providing further evidence that pure Nb grains are present in the films.

Spatial STM mapping

Density of states was obtained by STM dI/dV measurements at many points to determine the spatial uniformity of the superconducting properties. As shown in Figure S15, neither the DOS energy dependencies nor the gap magnitudes vary significantly in the center of a grain. Composition of Nb_3Sn can change significantly at grain boundaries, affecting the gap size and RF properties[8,9]; however, we did not perform DOS measurements at grain boundaries.

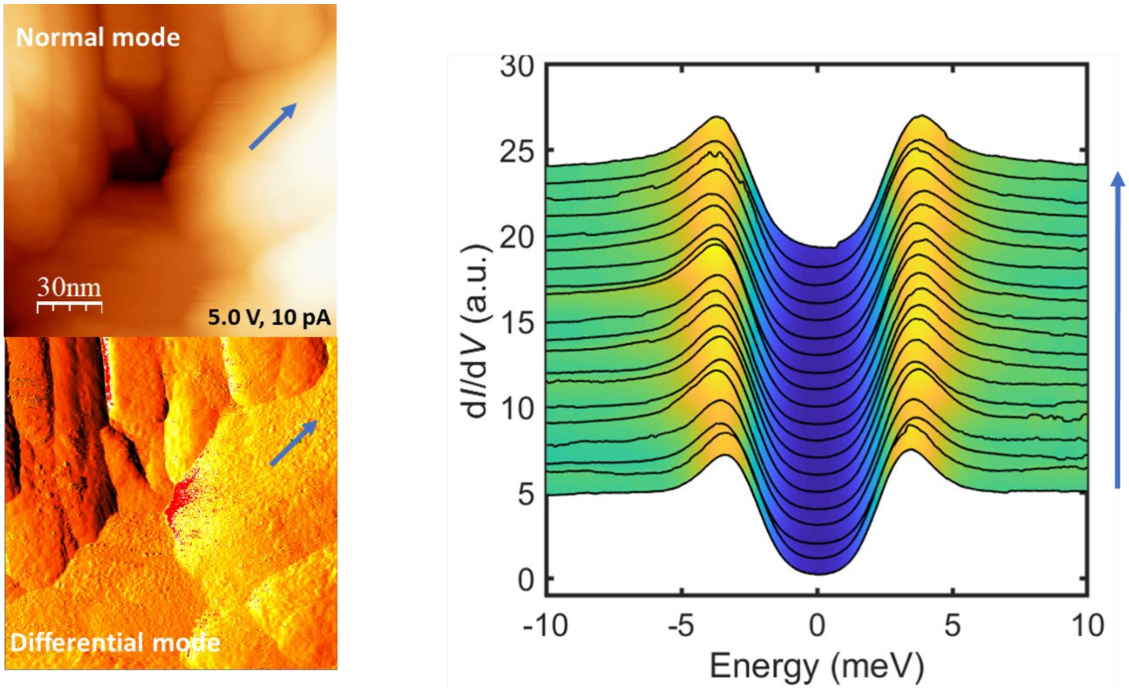

Figure S15: Density of states mapped across several points in a grain of Nb_3Sn grown on Nb-doped $SrTiO_3$. The arrows in the left figures show the regions, where the tunneling scans were measured.

RF Q_0 measurements in copper cavity

The low-field Q measurements reported in the main text were performed at power excitation levels of 1-10 mW. For the duty cycle of 10^{-6}, this translates to the RF field of about 30 μT on the surface of the sample.

In addition to the Nb-coated hemispherical cavity used for RF measurements, we have access to a cavity made of bare Cu. This setup can measure the Q factors of the sample at much higher fields. The Cu cavity does not have a critical magnetic field, making it possible to measure the quench fields higher than the quenching field of Nb. The Q_0 of the 120-nm Nb_3Sn film was measured in this Cu cavity at low fields. The result is plotted in Figure S16. Here Q_0(T) saturates below ~13 K, where the Cu cavity dominates RF losses. A hump in Q_0(T) at ~16.5 K corresponds to the transition of Nb_3Sn to the superconducting state. This indicates some inhomogeneity in the film, which is to be expected when there are Nb grains coexisting with Nb_3Sn in the film. The most important feature of this measurement is the strong dependence of Q_0 on the surface resistance of the Nb_3Sn. Above T_c of Nb_3Sn, Q_0 drops by a factor of ~100. These preliminary data assure that the transition of the film from superconducting to normal during high-field quench experiments will be easy to discern. This high-power setup can produce a peak power input up to 1 MW. For the duty cycle of 10^{-6}, the average RF power on the sample is about 1 W. At these power levels the peak RF fields on the sample surface can reach 360 mT.

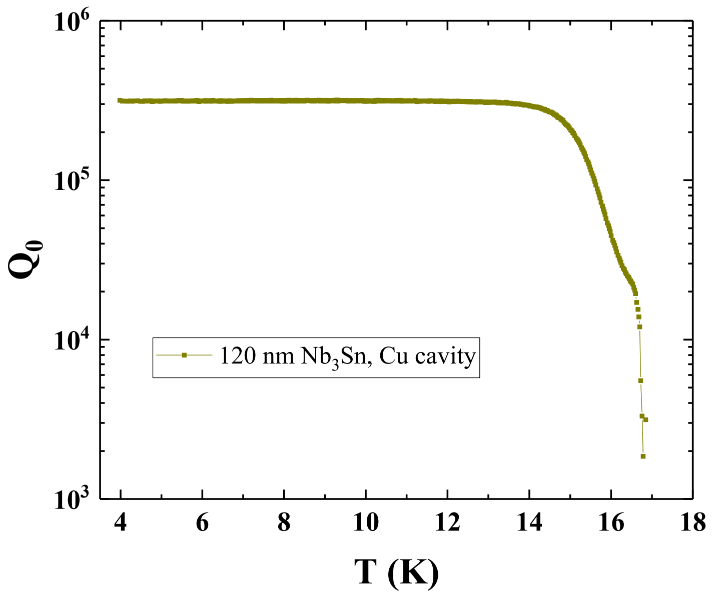

Figure S16: Low-power quality factor of 120 nm Nb3Sn film measured in Cu cavity. Low-T Q0 is limited by the Cu cavity. The differential in Q between normal and superconducting Nb3Sn is stark, a promising indication that quench fields will be very clear in high-field.

References

1. Maglić, K. D., Perovic, N. L., Vukovic, G. S. & Zekovic, L. P. Specific Heat and Electrical Resistivity of Niobium Measured by Subsecond Calorimetric Technique. *Int. J. Thermophys.* **15,** 963–972 (1994).

2. Wang, Q., Xiao, P., Yu, J. & Dai, J. Measuring normal spectral emissivities of niobium by a pulse-heating technique: 1000 K to the melting point. *Chinese Opt. Lett.* **04,** 701 (2006).

3. Gillet, M., Al Mohammad, A., Masek, K. & Gillet, E. Influence of surface structure on the growth of Au on αAl_2O_3 $(1\bar{1}012)$. *Thin Solid Films* **374,** 134–141 (2000).

4. Trainor, T. P., Eng, P. J., Brown, G. E., Robinson, I. K. & De Santis, M. Crystal truncation rod diffraction study of the α-Al2O3 (1 $\bar{1}$0 2) surface. *Surf. Sci.* **496,** 238–250 (2002).

5. Devantay, H., Jorda, J. L., Decroux, M. & Muller, J. The physical and structural properties of superconducting A15-type Nb- Sn alloys. *J. Mater.* **16,** 2145–2153 (1981).

6. Flukiger, R., Kuepfer, H., Jorda, J. & Muller, J. Effect of atomic ordering and composition changes on the R of Nb_3Al, Nb_3Sn, Nb_3Ge, Nb_3Ir, V_3Si and V_3Ga. *IEEE Trans. Magn.* **MAG-23,** 1596–1599 (1987).

7. Godeke, A. A review of the properties of Nb_3Sn and their variation with A15 composition, morphology and strain state. *Supercond. Sci. Technol.* **19,** R68–R80 (2006).

8. Lee, J. *et al.* Grain-boundary structure and segregation in Nb_3Sn coatings on Nb for high-performance superconducting radiofrequency cavity applications. *Acta Mater.* **188,** 155–165 (2020).

9. Lee, J. *et al.* Atomic-scale analyses of Nb 3 Sn on Nb prepared by vapor diffusion for superconducting radiofrequency cavity applications: a correlative study. *Supercond. Sci. Technol.* **32,** 024001 (2019).
